# *In vivo* CRISPR/Cas9 screens identify new regulators of B cell activation and plasma cell differentiation

**DOI:** 10.1084/jem.20250594

**Published:** 2026-01-28

**Authors:** Lesly Calderón, Markus Schäfer, Marina Rončević, René Rauschmeier, Markus Jaritz, Tanja A. Schwickert, Qiong Sun, Andrea Pauli, Johannes Zuber, Meinrad Busslinger

**Affiliations:** 1 Research Institute of Molecular Pathology (IMP), Vienna BioCenter, Vienna, Austria; 2 Institute of Allergy Research (IAF), Helmholtz Munich and Center of Allergy and Environment (ZAUM), Technical University of Munich, München, Germany; 3 Helmholtz Graduate School Environmental Health (HELENA), Munich, Germany

## Abstract

Immune responses to pathogens lead to the generation of plasma cells through a complex interplay of B cells with their microenvironment in lymphoid organs. To identify new regulators of B cell activation and plasmablast differentiation in the context of the splenic microenvironment, we established an *in vivo* system for pooled sgRNA CRISPR/Cas9 screens in immunized mice. To improve the infection efficiency of naïve B cells, we generated *Cd23*-Cre *Rosa26*^LSL-EcoR/+^ mice exhibiting increased expression of the ecotropic lentivirus receptor EcoR on naïve B cells. Upon adoptive B cell transfer and immunization of recipient mice, 379 sgRNAs, targeting genes with high expression in plasma cells, were analyzed for their effects on plasmablast generation. Gene hits, encoding 23 positive and 18 negative regulators of B cell activation, plasmablast differentiation, or homeostasis, were uniquely identified in these *in vivo* screens. Validated genes encoded proteins involved in cell adhesion, signal transduction, protein folding, iron transport, and enzymatic processes. Hence, our *in vivo* screening system identified novel regulators controlling B cell–mediated immune responses.

## Teaser

Immune responses to pathogens lead to the generation of plasma cells containing the infection by secreting high-affinity antibodies. Here, we describe an *in vivo* CRISPR/Cas9 screening system for identifying novel regulators of B cell activation and plasma cell development in the context of the splenic microenvironment of immunized mice.

## Introduction

B cell responses are essential for humoral immunity against pathogens as they lead to the generation of activated B cells and germinal center (GC) B cells that subsequently develop into antibody-secreting plasma cells that produce large amounts of antigen-specific antibodies to eliminate the infection (Cyster and Allen, 2019; Nutt et al., 2015). Upon antigen encounter, the newly generated, antibody-secreting cells in peripheral lymphoid organs remain proliferative and are thus referred to as plasmablasts. Upon withdrawal from the cell cycle, these plasmablasts can home to the bone marrow, where they develop into quiescent long-lived plasma cells, functioning as immunological memory against pathogens (Fooksman et al., 2024; Nutt et al., 2015; Tellier et al., 2024). Misguided B cell responses can also act as key mediators of diseases, such as allergies, antibody-mediated autoimmunity, and tumor malignancies (Cyster and Allen, 2019).

B cell responses are initiated in secondary lymphoid organs once mature B cells encounter their cognate antigens. The nature of the antigen determines how B cell responses develop and give rise to different outcomes (Elsner and Shlomchik, 2020). For instance, engagement of the B cell antigen receptor (BCR) by polysaccharide antigens (T cell–independent [TI] type II antigens) initiates a signaling cascade that is sufficient to cause B cell activation and subsequent differentiation into memory B cells and low-affinity antibody-secreting plasma cells (García de Vinuesa et al., 1999b; Obukhanych and Nussenzweig, 2006), whereas B cell activation by peptide antigens requires, in addition to BCR engagement, the interaction between B cells and cognate antigen-specific CD4^+^ T cells. These T cell–dependent (TD) B cell responses lead additionally to the generation of GCs within B cell follicles and the differentiation of GC B cells into memory B cells and plasma cells expressing higher affinity antibodies (Victora and Nussenzweig, 2022).

The transition from mature B cells to antibody-secreting cells is associated with substantial changes in cell morphology and the expression of hundreds of genes (Shi et al., 2015; Tellier et al., 2016; Minnich et al., 2016). Known key regulators of plasma cell differentiation and function are the transcription factors *Irf4* (Sciammas et al., 2006; Klein et al., 2006), Blimp1 (*Prdm1*) (Tellier et al., 2016; Minnich et al., 2016), *Xbp1* (Bettigole and Glimcher, 2015; Wöhner et al., 2022), and E-proteins (Wöhner et al., 2016; Gloury et al., 2016). Recent efforts to investigate the function of novel regulators expressed in plasma cells have used targeted CRISPR/Cas9 screens to inactivate many genes simultaneously (Newman and Tolar, 2021; Pinter et al., 2022; Trezise et al., 2023; Turner et al., 2022; Xiong et al., 2023; Chu et al., 2016). Although these studies have been comprehensive and revealed novel genes that are important for plasma cell differentiation, they were performed with *in vitro*–cultured activated B cells and, therefore, did not consider the role of the microenvironment in secondary lymphoid organs in controlling the development of B cell responses.

Immune cell activation, differentiation, and function are affected by complex cellular and microenvironmental signals that occur *in vivo* and cannot be easily modeled *in vitro*. Chemokines, cytokines, and adhesion molecules expressed by stromal cells or other hematopoietic cells in the niche have been shown to regulate B cell–mediated immune responses and the lifespan of plasma cells (Cassese et al., 2003; García de Vinuesa et al., 1999a; Hargreaves et al., 2001; Robinson et al., 2020). CRISPR/Cas9-based *in vivo* genetic screens in mouse models have recently been used to unravel the regulatory mechanisms of immune cell fate and function primarily in the T cell lineage (Dubrot et al., 2022; Huang et al., 2021; Long et al., 2021). To identify new regulators of B cell activation and plasma cell development in the context of the microenvironment of secondary lymphoid organs, we established an *in vivo* model system for pooled single-guide RNA (sgRNA) CRISPR/Cas9 screens. We performed successful screening experiments, in which naïve sgRNA-transduced donor B cells were transferred into recipient mice to undergo differentiation into activated B cells and plasma cells *in vivo* upon TD and TI immunization. Our screening experiments simultaneously investigated the functions of 379 genes that are highly expressed in plasma cells. Their importance in the development of B cell responses and in the generation, survival, and/or maintenance of antibody-secreting cells was tested. We identified and validated several novel positive and negative regulators that are implicated in the control of these late-stage immune processes.

## Results

### Establishing an *in vivo* model system for CRISPR/Cas9 screening of novel regulators of B cell responses

**Figure 1.**
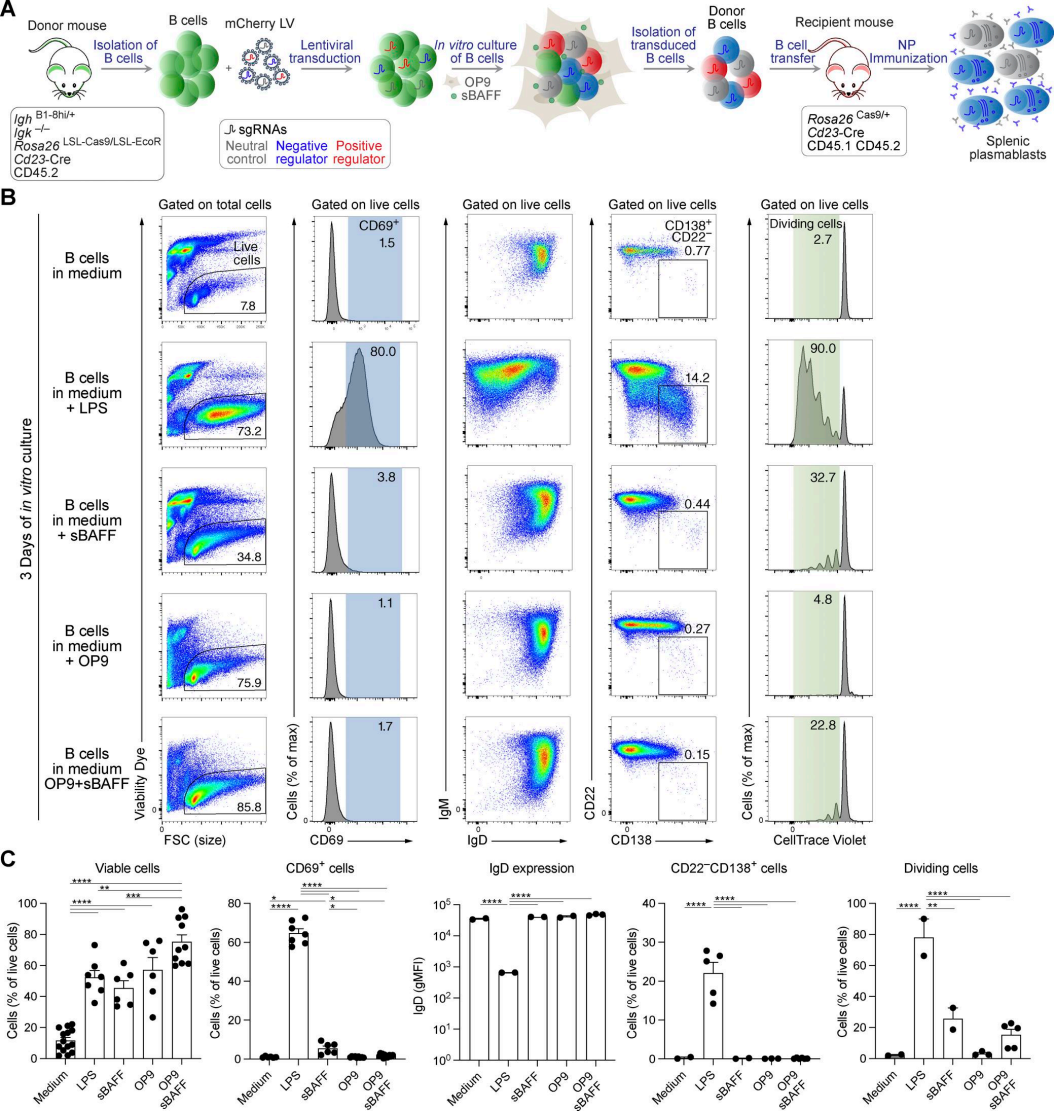
***In vitro* culture conditions maintaining the naïve B cell state. (A)** Schematic diagram of the different steps of the *in vivo* CRISPR/Cas9 screening system. Briefly, naïve B cells isolated from donor mice of the indicated genotype are transduced with LV particles expressing sgRNAs specific for the genes of interest and a neutral control gene. After infection, the cells are cultured *in vitro* for 3 days. Transduced mCherry^+^ donor B cells are sorted by flow cytometry and transferred to recipient mice of the indicated genotype, which are immunized with the hapten NP to induce an immune response. The resulting mCherry^+^ plasmablasts are isolated, and the abundance of sgRNAs in plasmablasts is determined and compared with the abundance of the sgRNAs in the donor B cells. **(B)** Flow cytometric analysis of wild-type naïve splenic B cells cultured *in vitro* for 3 days under different conditions. B cells were cultured either in the B cell medium alone, or additionally in the presence of LPS, sBAFF, OP9 cells, or OP9 cells plus sBAFF. Before starting the culture, B cells were labeled with the CellTrace Violet dye. After 3 days, the cell viability, CD69 and IgD cell surface protein expression, differentiation into plasmablasts (CD138^+^CD22^–^), and cell proliferation were assessed. **(C)** Summary of all the data generated with the different culture conditions indicated. The statistical data are shown as mean values with SEM and were analyzed by one-way ANOVA with Tukey’s multiple comparisons test; *P < 0.05; **P < 0.01; ***P < 0.001; ****P < 0.0001. Each dot corresponds to one mouse. Independent experiments were performed from two (IgD) to 10 (viability) times.

As the microenvironment of secondary lymphoid organs is crucial for the development of immune responses, we established an *in vivo* model system for CRISPR/Cas9 screens to identify new genes that regulate B cell–mediated immune responses. The system consists of isolating Cas9-expressing naïve B cells, transducing them with lentiviral (LV) particles carrying sgRNAs targeting the genes of interest, transferring these transduced naïve donor B cells to recipient mice, and evaluating the abundance of sgRNAs in the resulting plasmablasts after immunization of the recipient mice (Fig. 1 A).

As such, this system requires the transduction of naïve B cells and their *in vitro* culture for a few days, both of which are difficult to achieve without the activation of B cells. We first explored the *in vitro* culture conditions for naïve B cells by analyzing the viability and naïve status of B cells upon culturing. Thus, splenic B cells were cultured in B cell medium without any additional additives or in B cell medium containing lipopolysaccharide (LPS) as a reference culture condition. Additionally, B cells were cocultured with stromal OP9 cells in the presence or absence of soluble B cell–activating factor (sBAFF) or only with sBAFF. Among the conditions tested after 3 days, culturing of B cells on stromal OP9 cells alone or on OP9 cells with sBAFF appeared to be best in maintaining the naïve B cell state, as assessed by the absence of expression of the activation marker CD69, the maintenance of IgD expression, and the absence of plasmablasts (Fig. 1, B and C). The B cells cultured on OP9 cells with sBAFF exhibited the highest cell viability (75%) and contained on average 15% of dividing cells, while B cells cultured on OP9 cells alone contained no dividing cells, but had on average a lower cell viability (54%) (Fig. 1, B and C). For all further experiments, we have chosen the OP9 cell-plus-sBAFF condition, as we considered the higher cell viability to be more important for obtaining enough infected naïve B cells to be able to perform B cell transfer experiments.

**Figure 2.**
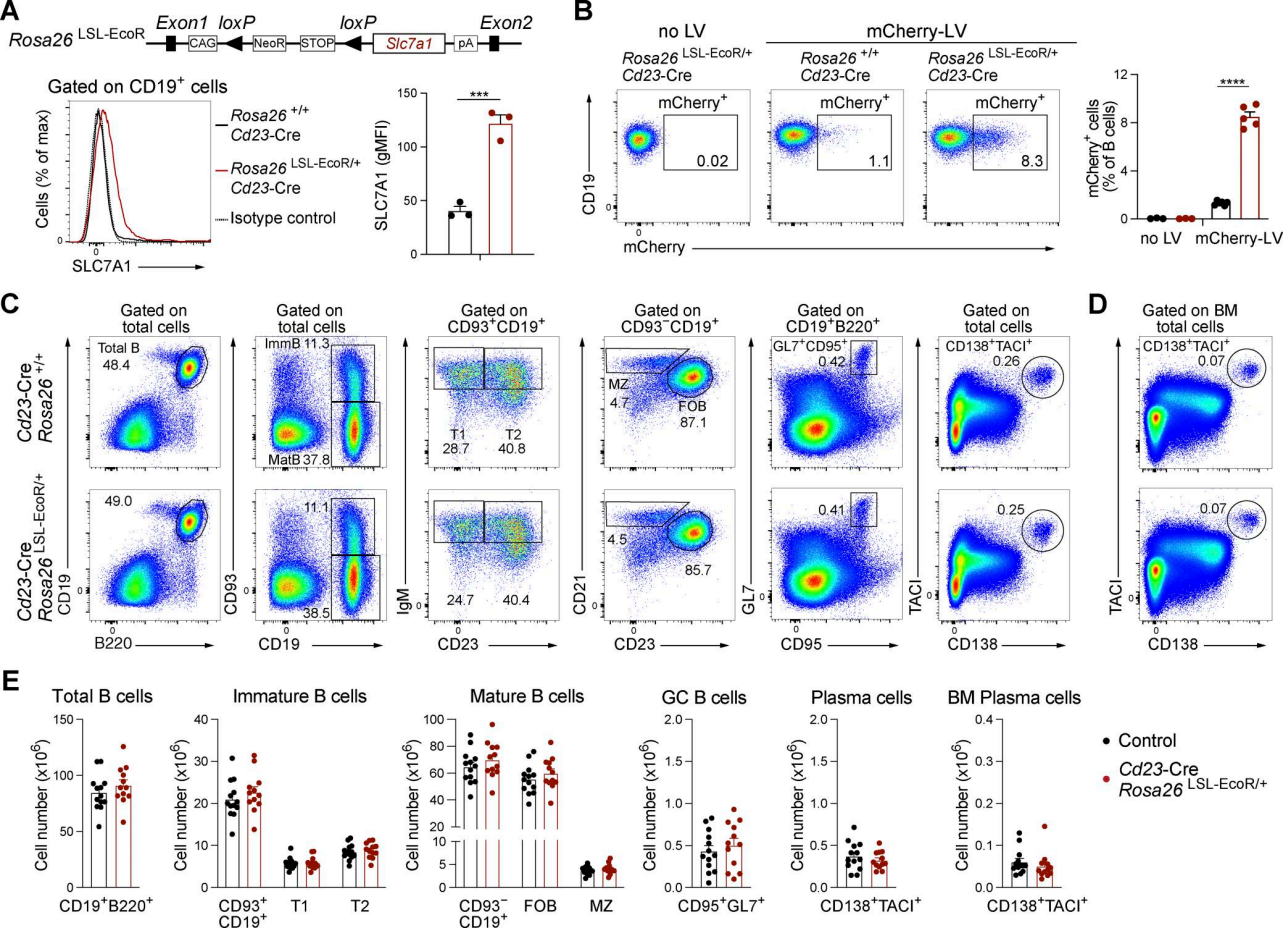
**Characterization of the *Rosa26*** ^**LSL-EcoR**^ **allele. (A)** Schematic diagram and expression of the *Rosa26*^LSL-EcoR^ allele. SLC7A1 (EcoR) protein expression was analyzed by flow cytometry in mature B cells from *Cd23*-Cre *Rosa26*^LSL-EcoR/+^ and control *Cd23*-Cre *Rosa26*^+/+^ mice and is displayed as a histogram or quantification of the geometric mean fluorescence intensity (gMFI). Statistical data are shown as mean values with SEM and were analyzed by the unpaired *t* test; ***P < 0.001. Each dot represents one mouse. **(B)** Flow cytometric analysis of B cells from *Cd23*-Cre *Rosa26*^LSL-EcoR/+^ and control *Cd23*-Cre *Rosa26*^+/+^ mice, which were transduced with ecotropic LV particles expressing the mCherry fluorescent reporter protein (mCherry-LV) and were subsequently cultured *in vitro* for 3 days in the presence of OP9 cells with sBAFF. The percentage of transduced mCherry^+^ B cells is shown. The bar graph indicates the transduction efficiency relative to all B cells. Statistical data are shown as mean values with SEM and were analyzed by the multiple unpaired *t* test with Holm–Šídák’s correction; ****P < 0.0001. Two experiments were performed, and each dot corresponds to one mouse. **(C)** Flow cytometric analysis of splenic B cells from *Cd23*-Cre *Rosa26*^LSL-EcoR/+^ and control *Cd23*-Cre *Rosa26*^+/+^ mice. The frequencies are shown for total B cells (CD19^+^B220^+^), immature B cells (CD19^+^CD93^+^), T1 B cells (CD19^+^CD93^+^IgM^hi^CD23^−^), T2 B cells (CD19^+^CD93^+^IgM^hi^CD23^+^), mature B cells (CD19^+^CD93^−^), MZ B cells (CD19^+^CD93^−^CD21^hi^Cd23^−/lo^), FO B cells (CD19^+^CD93^−^CD21^lo^CD23^hi^), GC B cells (CD19^+^B220^+^GL7^+^CD95^+^), and plasma cells (CD138^+^TACI^+^). **(D)** Flow cytometric analysis of bone marrow plasma cells from *Cd23*-Cre *Rosa26*^LSL-EcoR/+^ and control *Cd23*-Cre *Rosa26*^+/+^ mice. **(E)** Quantification of the number of total B, immature B, mature B, GC B, and plasma cells in the spleen, and plasma cells in the bone marrow from *Cd23*-Cre *Rosa26*^LSL-EcoR/+^ and control (*Cd23*-Cre *Rosa26*^+/+^ and *Rosa26*^+/+^) mice. Statistical data are shown as mean values with SEM. All pairwise comparisons are nonsignificant, as analyzed by the multiple unpaired *t* test with Holm–Šídák’s correction test. Two (A and B) and three (C–E) independent experiments were performed. Each dot represents one mouse.

**Figure S1.**
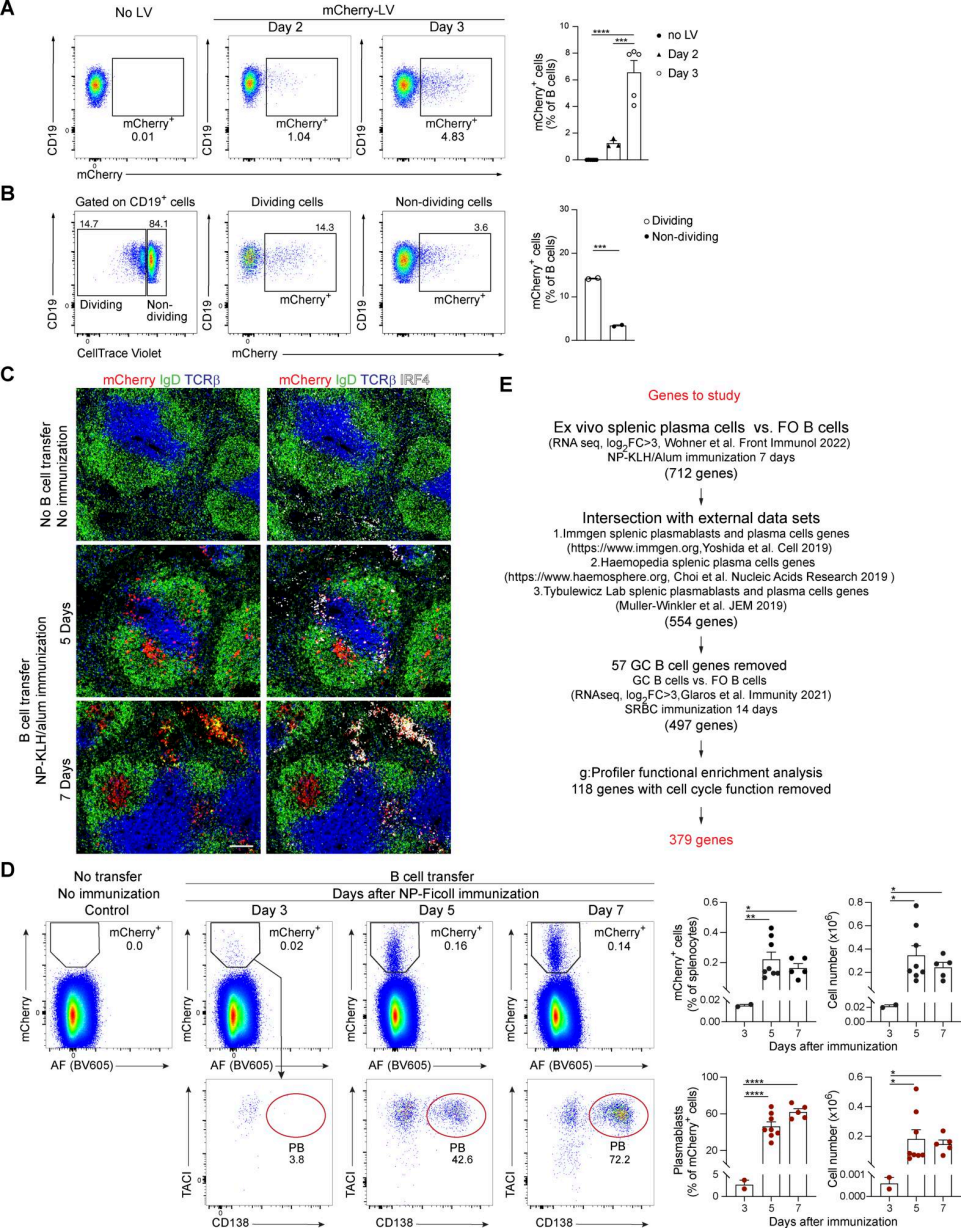
**Description and characterization of different aspects of the sgRNA screening system. (A)** Flow cytometric analysis of mCherry expression in B cells at 2 and 3 days after infection with a control mCherry-LV. The cells were cultured on OP9 cells with sBAFF (right). The frequencies of mCherry^+^ B cells are shown as mean values with SEM and were analyzed by one-way ANOVA with Tukey’s multiple comparisons test; ***P < 0.001; ****P < 0.0001. **(B)** Flow cytometric analysis of dividing and nondividing mCherry^+^ B cells after 3 days in culture on OP9 cells with sBAFF. Mature CD43^–^ B cells were incubated with CellTrace Violet prior to infection with a control mCherry-LV and were subsequently cultured for 3 days. The frequencies of mCherry^+^ B cells among the dividing and nondividing B cell population are shown as mean values with SEM and were analyzed by the unpaired *t* test; ***P < 0.001. **(C)** Immunofluorescence staining of spleen sections from recipient mice at 5 and 7 days after immunization with NP-KLH/alum. LV infection and B cell transfer were performed as described in D. Spleen sections were stained with antibodies against IgD (green), mCherry (red), TCRβ (blue), and IRF4 (white). A nonimmunized mouse, which did not receive transduced mCherry^+^ B cells, was used as a control. The scale bar represents 100 μm. **(D)** Time course of plasmablast formation upon immunization with NP-Ficoll. Naïve B cells isolated from the donor mice were transduced with LV particles expressing a neutral control sgRNA (sg.*Chr1*) and the mCherry reporter protein. After infection, B cells were cultured *in vitro* for 3 days in the presence of stromal OP9 cells and sBAFF. Transduced mCherry^+^ B cells were isolated and transferred to recipient mice, which were immunized 16 h later with NP-Ficoll in PBS. Flow cytometric analysis of splenocytes from recipient mice at day 3, 5, and 7 after immunization (left) revealed the percentages of mCherry^+^ B cells in total splenocytes and mCherry^+^ plasmablasts (TACI^+^CD138^+^) within the mCherry^+^ cells, as shown in the bar graphs (right). Absolute numbers of the mCherry^+^ B cells and mCherry^+^ plasmablasts in the spleen are also indicated (far right). Statistical data are shown as mean values with SEM and were analyzed by the Welch ANOVA with the Brown–Forsythe test; *P < 0.05; **P < 0.01; ****P < 0.0001. Each dot represents one mouse. AF, autofluorescence measured in the BV605 channel. **(E)** Scheme describing the steps taken for the selection of the 379 genes to be studied in the *in vivo* CRISPR/Cas9 screening experiments. The data shown in A, B, and D are based on two independent experiments. Each dot corresponds to one mouse.

Unless activated, naïve B cells are poorly transducible with commonly used LVs. This poor infection efficiency is likely caused by the low expression of the corresponding viral receptors on naïve B cells. We therefore used CRISPR/Cas9 engineering to generate a *Rosa26* knock-in mouse that carries the cDNA encoding the solute carrier SLC7A1 downstream of a *lox*P-flanked transcriptional termination sequence (*Lox*P-Stop-*Lox*P [LSL]) (Fig. 2 A). SLC7A1 is the receptor for ecotropic LVs, which is also known as ecotropic receptor (EcoR). Mice carrying the *Rosa26*^LSL-EcoR^ allele were crossed with transgenic *Cd23*-Cre mice (Kwon et al., 2008) to generate *Cd23*-Cre *Rosa26*^LSL-EcoR/+^ mice. Cre recombinase expression from the *Cd23*-Cre transgene (Kwon et al., 2008) is initiated during the transition from immature to mature B cells, thus leading to excision of the stop cassette and transcription of the *Slc7a1* cDNA in mature B cells. Flow cytometric analysis showed that splenic B cells isolated from *Cd23*-Cre^LSL-EcoR/+^ mice expressed threefold higher levels of the EcoR SLC7A1 compared with B cells from control *Cd23*-Cre *Rosa26*^+/+^ mice (Fig. 2 A). B cells isolated from these mice were infected *ex vivo* with ecotropic LV particles expressing mCherry as a fluorescent reporter protein (mCherry-LV) (Fig. 2 B). After infection and 3 days of *in vitro* culture on OP9 cells with sBAFF, B cell transduction was assessed by flow cytometry. Approximately 8% of the B cells from *Cd23*-Cre *Rosa26*^LSL-EcoR/+^ mice were mCherry^+^, whereas control B cells isolated from *Cd23*-Cre *Rosa26*^+/+^ mice were poorly transduced, as expected (Fig. 2 B). Notably, the percentage of infected mCherry^+^ B cells strongly increased from day 2 to day 3 during *in vitro* culture on OP9 cells with sBAFF (Fig. S1 A), while the mCherry-LV apparently infected the few dividing cells in culture with a fourfold higher efficiency compared with the majority of nondividing cells (Fig. S1 B). We therefore sorted the infected mCherry^+^ B cells after 3 days in culture, followed by their transfer to recipient mice.

As SLC7A1 is a cationic amino acid transporter, its higher expression could affect B cell differentiation. By flow cytometric analysis, we therefore assessed the percentage and number of different B cell populations in *Cd23*-Cre *Rosa26*^LSL-EcoR/+^ and control *Cd23*-Cre *Rosa26*^+/+^ or *Rosa26*^LSL-EcoR/+^ mice at the steady state. No differences were found in the percentage or number of total B, immature B, transitional 1 (T1) B and transitional 2 (T2) B, mature B, follicular (FO) B, marginal zone (MZ) B, and GC B cells and plasmablasts in the spleen, or bone marrow plasma cells (Fig. 2, C–E). Hence, we conclude that ectopic expression of SLC7A1 does not affect late B lymphopoiesis.

The design of the *in vivo* model system for CRISPR/Cas9 screening depends on donor B cells from mice carrying the *Rosa26*^LSL-EcoR^ and *Rosa26*^LSL-Cas9^ alleles, as well as the *Cd23*-Cre transgene (Fig. 1 A). To exclude differentiation biases due to differences in BCR affinity or specificity, we used B cells from mice carrying the immunoglobulin heavy chain *Igh*^B1-8hi^ and immunoglobulin κ light-chain gene knockout (*Igk*^−^) alleles, which generate monoclonal B cells expressing the same BCR (Fig. 1 A). The *Igh*^B1-8hi^ allele expresses a rearranged immunoglobulin heavy chain that, when combined with an immunoglobulin λ light chain, recognizes the hapten 4-hydroxy-3-nitrophenyl acetyl (NP) (Shih et al., 2002). Hence, the genotype of the donor mice is *Cd23*-Cre *Rosa26*^LSL-EcoR/LSL-Cas9^*Igh*^B1-8hi/+^*Igk*^–/–^ (Fig. 1 A). Approximately 95% of the B cells in these mice are specific for the hapten NP. Once isolated, infected with ecotropic LVs, and cultured *in vitro* for 3 days on OP9 cells with sBAFF, the transduced B cells expressing a fluorescent protein will be isolated by flow cytometric cell sorting and transferred to recipient mice. Recipient mice of the *Cd23*-Cre *Rosa26*^Cas9/+^ genotype express the immunogenic Cas9 endonuclease and Cre recombinase and thus tolerate the donor B cells (Fig. 1 A). After the transfer of donor B cells, recipient mice will be immunized with the antigen NP, and several days later, donor B cell–derived plasmablasts will be isolated, and the abundance of sgRNAs in the plasmablast population in comparison with the abundance of sgRNAs in the donor B cells (before transfer) will be determined. Underrepresented and overrepresented sgRNAs are considered to target potentially positive and negative regulators of B cell activation or plasmablast differentiation, respectively (Fig. 1 A).

### Validation of the *in vivo* CRISPR/Cas9 screening system

**Figure 3.**
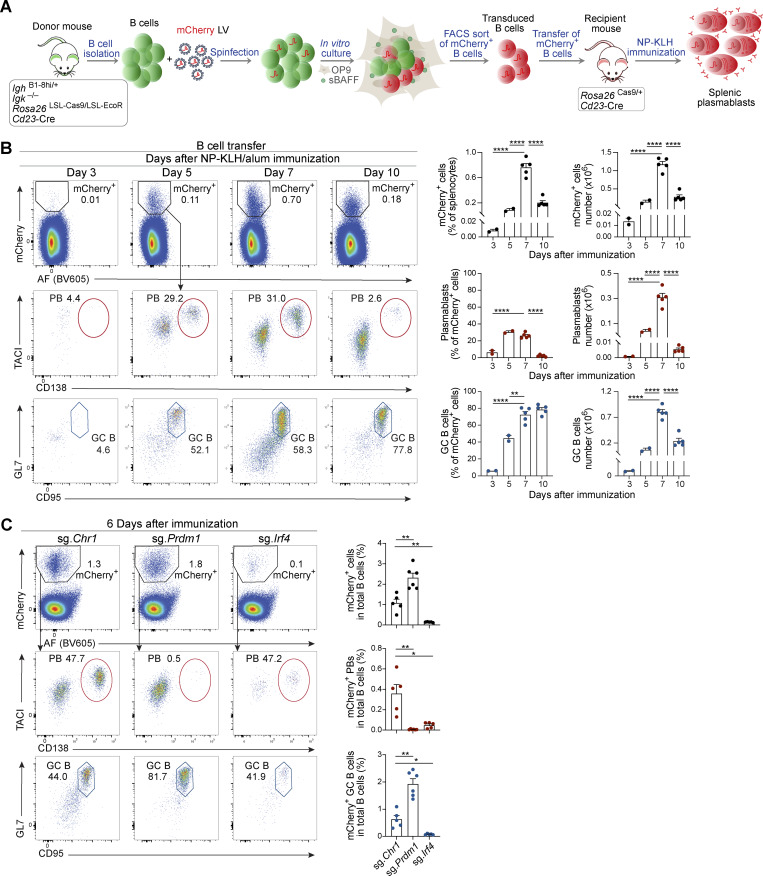
**Testing of the *in vivo* model system used for CRISPR/Cas9 screening experiments. (A)** Schematic diagram of the setup used for testing the *in vivo* CRISPR/Cas9 screening system. Naïve B cells isolated from donor mice were transduced with LV particles expressing a neutral control sgRNA and the mCherry reporter protein. After infection, B cells were cultured *in vitro* for 3 days in the presence of OP9 cells and sBAFF. Transduced mCherry^+^ B cells were sorted by flow cytometry and transferred to recipient mice, which were immunized 16 h later with NP-KLH in alum. mCherry^+^ plasma cells and mCherry^+^ GC B cells were analyzed by flow cytometry at different time points after immunization. **(B)** Naïve B cells of the donor genotype were infected with a mCherry-LV expressing a neutral control sgRNA and sorted followed by transfer to mice of the recipient genotype and NP-KLH immunization, as described in A. Flow cytometric analysis of splenocytes from recipient mice was performed 3, 5, 7, and 10 days after immunization. The percentages of total mCherry^+^ B cells in total splenocytes, mCherry^+^ plasmablasts (TACI^+^CD138^+^), and mCherry^+^ GC B cells (GL7^+^CD95^+^) within the mCherry^+^ B cell population (left), and the absolute numbers of these cells (right) are indicated. AF, autofluorescence measured in the BV605 channel. **(C)** Naïve B cells isolated from donor mice were transduced with LV particles expressing a control sgRNA (sg.*Chr1*) or sgRNAs targeting *Prdm1* or *Irf4*. After infection, B cells were cultured *in vitro* for 3 days and transduced mCherry^+^ B cells were transferred to recipient mice followed by immunization with NP-KLH, as described in A. 6 days after immunization, the splenocytes were analyzed by flow cytometry. The percentages of total mCherry^+^ B cells, mCherry^+^ plasmablasts, and mCherry^+^ GC B cells among total B cells are indicated. Statistical data (B and C) are shown as mean values with SEM and were analyzed by the multiple unpaired *t* test with Holm–Šídák’s correction test; *P < 0.05; **P < 0.01; ****P < 0.0001. Two independent experiments (B and C) were performed. Each dot represents one mouse.

After setting up the different steps of the screening protocol, we next tested the functionality of this *in vivo* model system (Fig. 3 A). For this, naïve B cells were isolated from donor mice and transduced with LV particles expressing mCherry as a fluorescent reporter protein and a neutral control sgRNA (sg.*Chr1*, also referred to as sg.Control, Table S1) targeting a sequence in a gene desert region on *Chr1*. Approximately 16 h after transfer, recipient mice were immunized with NP-conjugated keyhole limpet hemocyanin (NP-KLH)/alum to induce a TD B cell immune response. On different days after immunization, we investigated the presence and fate of splenic mCherry^+^ donor-derived B cells by flow cytometric analysis (Fig. 3 B). At day 3 after immunization, 13,400 mCherry^+^ cells were detected, which did not yet differentiate into plasmablasts or GC B cells (Fig. 3 B). In contrast, at day 5 and 7 after immunization, the spleen contained higher proportions of mCherry^+^ cells, which consisted of plasmablasts (30%) and GC B cells (52–58%). At day 10, the proportion of mCherry^+^ cells was already reduced again with most of the remaining cells being GC B cells and only very few cells being plasmablasts (Fig. 3 B). Immunofluorescence staining of spleen sections from recipient mice at 5 and 7 days after NP-KLH immunization revealed mCherry^+^ IRF4^+^ plasmablasts and mCherry^+^ IgD^–^ GC B cells located in the splenic red pulp or B cell follicles, respectively (Fig. S1 C).

As a proof of principle, we next tested the *in vivo* system by transducing naïve donor B cells with LV particles carrying sgRNAs targeting the genes of the well-known plasma cell regulators *Irf4* (Mittrücker et al., 1997; Sciammas et al., 2006) and *Prdm1* (Blimp1) (Shapiro-Shelef et al., 2003; Turner et al., 1994). Recipient mice received mCherry^+^ donor B cells, which expressed either sg.*Prdm1*, sg.*Irf4*, or sg.*Chr1*, followed by immunization with NP-KLH/alum and flow cytometric analysis at day 6 after immunization (Fig. 3 C). As expected, mCherry^+^ plasmablasts and mCherry^+^ GC B cells were generated in the control sg.*Chr1* experiment. In contrast, the sg.*Prdm1* experiment exhibited no mCherry^+^ plasmablasts, but an increased proportion of mCherry^+^ GC B cells, indicating that *Prdm1* inactivation in donor B cells led to GC B cell accumulation (Fig. 3 C). Almost no mCherry^+^ B cells were found in the spleen of mice that received donor B cells expressing sg.*Irf4* (Fig. 3 C), as expected, because activated B cells are known to require IRF4 to differentiate into GC B cells and plasma cells (Ochiai et al., 2013; Willis et al., 2014).

We next investigated the utility of the *in vivo* CRISPR/Cas9 system also for studying TI B cell immune responses. Recipient mice receiving mCherry^+^ donor B cells were immunized with the NP-conjugated polysaccharide Ficoll (NP-Ficoll) in PBS (Fig. S1 D). Flow cytometric analysis of splenocytes identified 21,600 mCherry^+^ cells on day 3 after immunization but revealed increased numbers of mCherry^+^ cells on days 5 and 7. At day 7, 70% of the mCherry^+^ cells were plasmablasts (Fig. S1 D).

Together, these results demonstrated that the novel *in vivo* CRISPR/Cas9 system was effective and functional, thus indicating its suitability for pooled sgRNA-based screens *in vivo* in mature B cells.

### Identification of new regulators of B cell responses by *in vivo* screens

**Figure S2.**
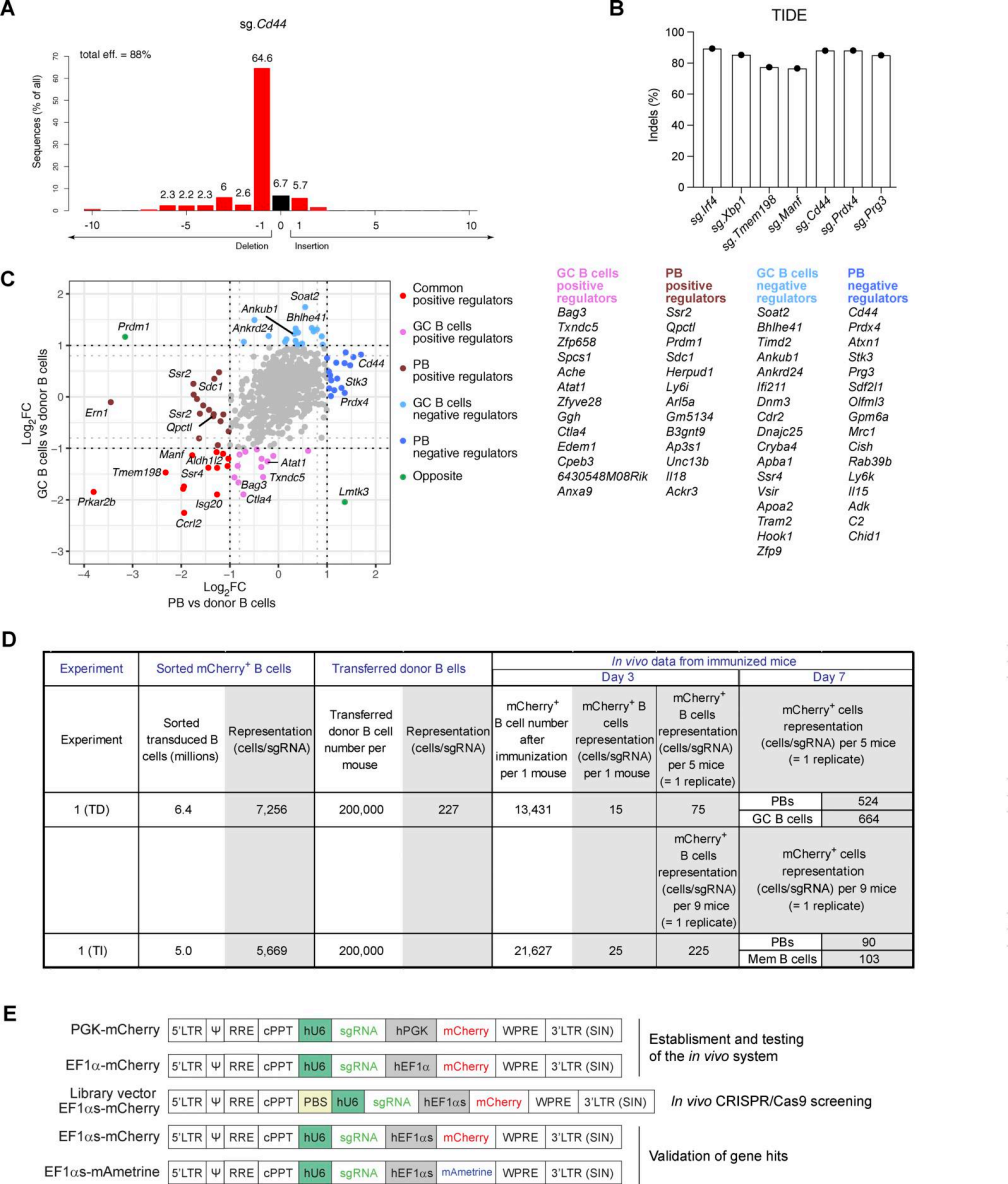
**Indel sequencing data, identification of GC B cell– and PB-specific sgRNA hits, and sgRNA representation at different stages of the screening experiments. (A and B)** Indel sequencing. Mature CD43^–^ B cells were infected with the indicated sgRNA mCherry-LVs, cultured for 3 days on OP9 cells with sBAFF, and then stimulated with CpG, IL-4, and IL-5 for another 3 days, followed by flow cytometric sorting of the mCherry^+^ B cells and DNA preparation. Indel sequencing was performed by PCR amplification and sequencing of a DNA fragment spanning the sgRNA break site, followed by TIDE analysis (Brinkman et al., 2014). **(A)** Percentages of indels are indicated relative to the break site of sg.*Cd44* (position 0, black), demonstrating that the percentage of indels is 88% for the sg.*Cd44*. **(B)** Percentages of indels are shown for the indicated sgRNAs, as determined by the indel sequencing and TIDE analysis. **(C)** Identification of GC B cell–specific and PB-specific positive and negative regulators, as determined by CRISPR/Cas9 screening experiments at day 7 after NP-KLH immunization. The log_2_FC plot (left) indicates the sgRNA hits that were determined by a more than twofold change in sgRNA abundance in GC B cells and PBs (corresponding to Fig. 4, B–E). The genes corresponding to the GC B cell–specific and PB-specific sgRNA hits are shown (right), and their fold changes and P values are indicated in Table S3. The common positive regulators are shown in Fig. 4, C and D, as being significant in both cell types, while the PB-specific positive regulators are indicated as being nonsignificant in the GC B cell analysis (Fig. 4, C and D). **(D)** sgRNA representation at different stages of the TD and TI screening experiments. Flow cytometric analysis was used to determine the number of sorted mCherry^+^ B cells at the start, the splenic mCherry^+^ B cells at day 3 after B cell transfer (Fig. 3 B [TD] and S1D [TI]), and the splenic mCherry^+^ PBs, GC B cells, and memory B cells at day 7 (Figs. S3 D [TD] and S4D [TI]). As the screening library contained 882 sgRNAs, the number of identified B cells was divided by 882 to determine how many cells contained one specific sgRNA (cells/sgRNA) under the assumption that each cell was only infected by one sgRNA virus (MOI = 1). **(E)** Schematic representation of the LV vectors used in this study. LVs containing the PGK-mCherry or EF1a-mCherry gene were used for establishing and testing of the *in vivo* CRISPR/Cas9 screening system. The sgRNA library was cloned in a LV vector containing the EF1as-mCherry gene. The validation experiments were performed with LVs containing the EF1as-mCherry or EF1as-mAmetrine gene. 5′LTR, 5′ long terminal repeat; Ψ, psi packaging signal; RRE, Rev response element; cPPT, central polypurine tract; PBS, primer binding site for DNA sequencing library preparation; hU6, human U6 promoter; hPGK, human phosphoglycerate kinase promoter; hEF1a, human elongation factor 1a promoter; hEF1as, human elongation factor 1a short promoter; WPRE, woodchuck hepatitis virus posttranscriptional regulatory element; 3′LTR (SIN), 3′ long terminal repeat (self-inactivating); FC, fold change; PBs, plasmablasts.

**Figure S3.**
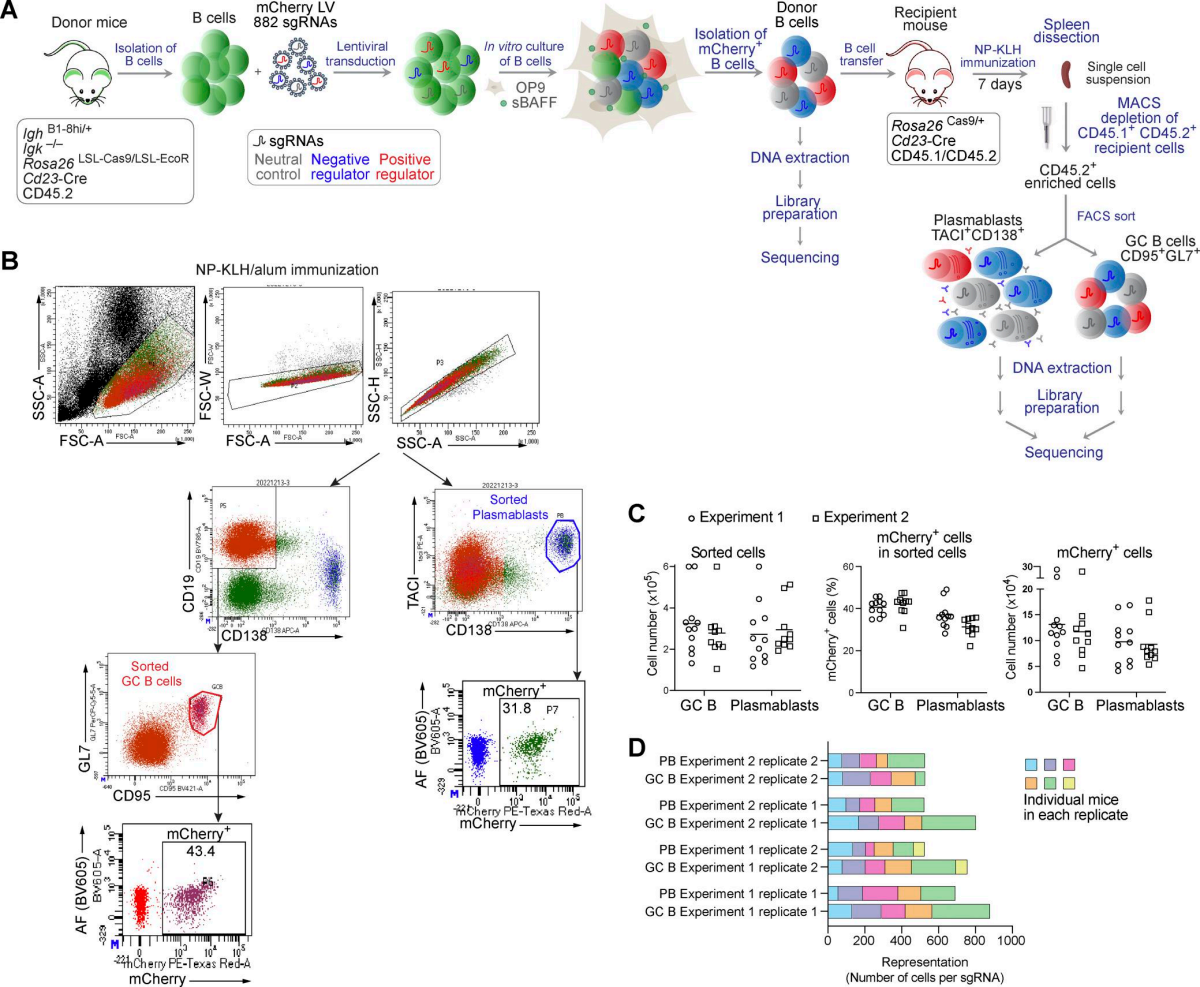
**CRISPR/Cas9 screen for identifying new regulators of the TD B cell responses. (A)** Schematic diagram of the *in vivo* CRISPR/Cas9 screening experiments used to study the TD B cell responses. Naïve B cells isolated from donor mice were transduced with LV particles, each expressing one of the 882 sgRNAs and the mCherry reporter protein. After infection, B cells were cultured *in vitro* for 3 days in the presence of OP9 cells and sBAFF. Transduced mCherry^+^ donor B cells were sorted by flow cytometry and transferred to recipient mice, which were immunized 16 h later with NP-KLH/alum. 7 days after immunization, splenic mCherry^+^ CD45.2^+^ cells were enriched by anti-CD45.1 antibody–mediated MACS depletion of the CD45.1^+^CD45.2^+^ recipient cells. Plasmablasts and GC B cells were isolated from the enriched fraction by flow cytometric sorting, followed by DNA extraction, library preparation, and DNA sequencing. DNA was also extracted from a fraction of the sorted transduced mCherry^+^ donor B cells before immunization, followed by library preparation and DNA sequencing. **(B)** Flow cytometric analysis of the enriched mCherry^+^ CD45.2^+^ B cells from recipient mice. The gates used for the sorting of plasmablasts (TACI^+^CD138^+^) and GC B cells (CD19^+^GL7^+^CD95^+^) are indicated. The percentages of mCherry^+^ plasmablasts and mCherry^+^ GC B cells within the sorted cell population are shown. **(C)** Numbers of sorted plasmablasts and GC B cells (left), the percentage of mCherry^+^ plasmablasts and mCherry^+^ GC B cells within the sorted cell population (middle), and the numbers of mCherry^+^ plasmablasts and mCherry^+^ GC B cells (right) were determined for individual recipient mice analyzed in two independent screening experiments. **(D)** Total sgRNA representation in each plasmablast and GC B cell replicate sample analyzed in two screening experiments. The sgRNA representation was estimated by dividing the number of mCherry^+^ plasmablasts or GC B cells by the number of the 882 sgRNAs constituting the sgRNA library. Each plasmablast and GC B cell replicate sample contained cells that were obtained from five or six recipient mice after pooling. Each colored box represents the individual contribution of each mouse to the sgRNA representation of the entire replicate sample.

As genome-wide sgRNA CRISPR/Cas9 screens are not feasible *in vivo* in the mouse, we performed instead pooled sgRNA screens. For this purpose, we selected genes, which are highly expressed in plasma cells compared with naïve B cells. Analysis of published RNA-seq data from our laboratory revealed 712 genes with a more than eightfold higher expression in plasma cells (Wöhner et al., 2022) (Fig. S1 E). We next removed genes, which were not present in at least one of three similar RNA sequencing (RNA-seq) datasets from other laboratories (Müller-Winkler et al., 2020; Choi et al., 2019; Yoshida et al., 2019), were highly expressed in GC B cells (Glaros et al., 2021), or were associated with cell proliferation, which resulted in 379 selected genes (Fig. S1 E). We then chose two sgRNAs for each gene of interest, which were designed according to the VBC score sgRNA prediction tool (Michlits et al., 2020), that is explained in detail in the Materials and methods. Insertion and deletion (indel) sequencing analysis of selected sgRNAs revealed a high editing efficiency of 80–90% of indels, as shown in Fig. S2, A and B. The final sgRNA library contained 882 sgRNAs including two sgRNAs of 62 control genes (Table S2 and Materials and methods). We next prepared a library of ecotropic mCherry LVs carrying the 882 sgRNAs for subsequent infection of naïve donor B cells (Fig. S3 A).

**Figure 4.**
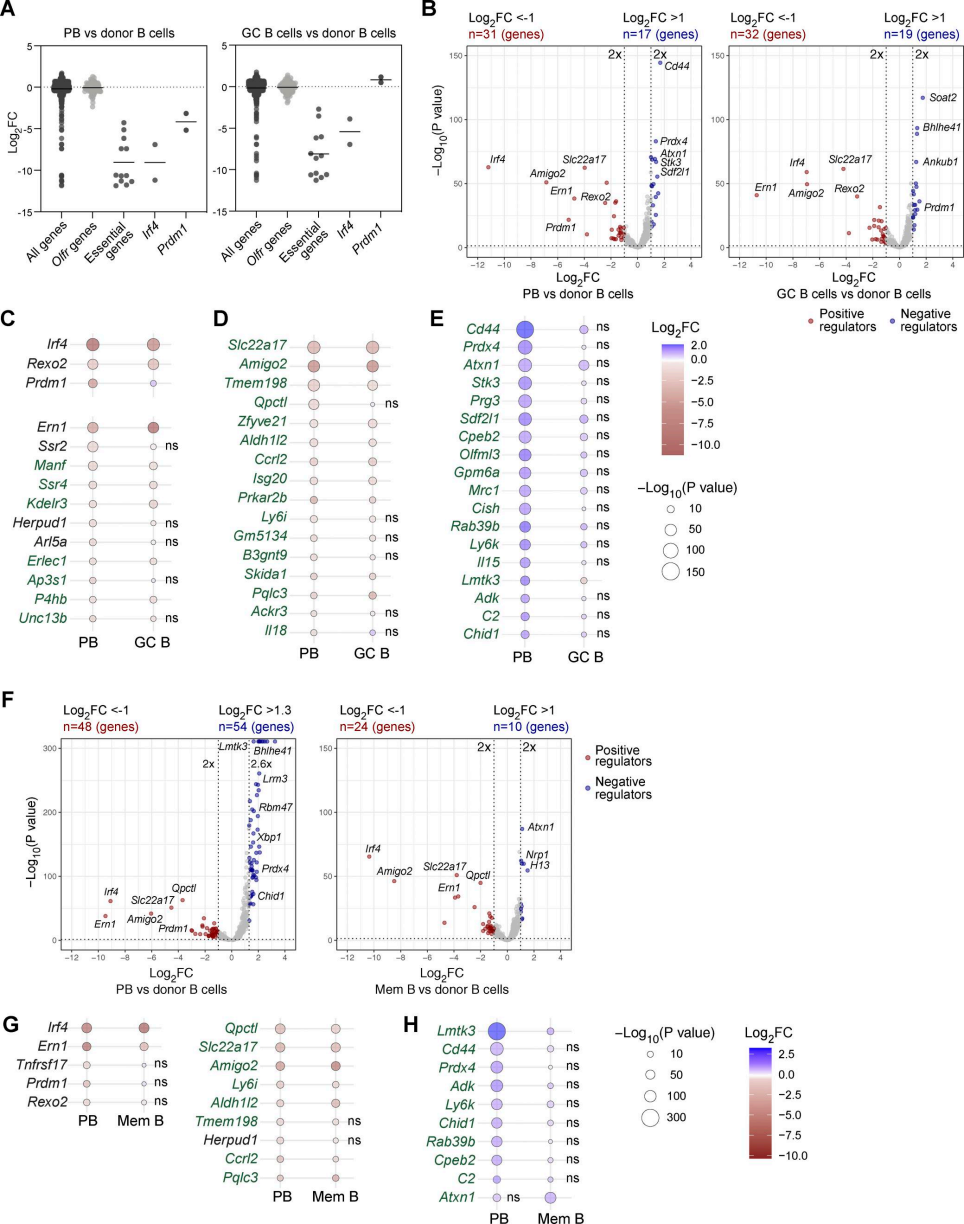
**CRISPR/Cas9 screens for new regulators of B cell responses. (A–E)** Results of the CRISPR/Cas9 screens performed upon TD immunization. **(A)** Difference of sgRNA abundance in PBs versus donor B cells (left) and GC B cells versus donor B cells (right) is shown as log_2_ FC for all sgRNAs in the library, neutral control sgRNAs targeting olfactory receptor genes (*Olfr*), sgRNAs targeting essential genes, and sgRNAs targeting the well-known PB regulators *Irf4* and *Prdm1*. **(B)** Volcano plot displaying the depletion or enrichment of sgRNAs (x axis) in PBs versus donor B cells (left) and in GC B cells versus donor B cells (right). The P values (y axis) were calculated using MAGeCK (Li et al., 2014). For each gene, the sgRNA with the more significant P value was plotted. A twofold change in sgRNA abundance is indicated by a dashed line. Potentially positive (brown, P < 0.05, log_2_FC ≤ −1) and negative (blue, P < 0.05, log_2_FC ≥ 1) regulators are highlighted for PBs (left plot) and GC B cells (right plot). **(C–E)** Heat map showing the depletion or enrichment of sgRNAs in PBs versus control donor B cells (left column) and in GC B cells versus control donor B cells (right column). The different color shadings indicate the log_2_FC, while the circle size refers to the P value (−log_10_). Genes uniquely found in our *in vivo* screens for PB regulators are highlighted in green color. *Erlec1, Qpctl*, *Zfyve21*, and *Isg20* were previously identified in an *in vitro* CRISPR/Cas9 screen as regulators of antibody secretion, but not as regulators of PB differentiation (Trezise et al., 2023). The sgRNA depletion data for well-known positive regulators of plasma cell development (above) and positive regulators involved in the homeostasis of the ER (below) are shown in C. The sgRNA depletion data of novel potentially positive regulators of PB differentiation or homeostasis are shown in D. The sgRNA enrichment data for novel potentially negative regulators of PB differentiation or homeostasis are shown in E. ns, nonsignificant. **(F–H)** Results of the CRISPR/Cas9 screens performed upon TI immunization. **(F)** Volcano plot displaying the depletion or enrichment of sgRNAs (x axis) in PBs versus donor B cells (left) and memory B cells (Mem B, TACI^+^CD138^−^) versus donor B cells (right). The P values (y axis) were calculated using MAGeCK. For each gene, the sgRNA with the more significant P value was plotted. Potentially positive (brown, P < 0.05 and log_2_FC ≤ −1) and negative (blue, P < 0.05 and log_2_FC ≥ 1) regulators are highlighted for PBs (left) and memory B cells (right). **(G and H)** Common potential PB regulators identified in the TI and TD CRISPR/Cas9 screening experiments. The heat maps show the depletion (G) or enrichment (H) of the sgRNAs, which were identified in the TI CRISPR/Cas9 screen by comparing PBs versus control donor B cells (left column) and memory B cells versus control donor B cells (right column). The sgRNA depletion data for well-known positive regulators of plasma cell development (left) and novel potentially positive regulators of PB differentiation or homeostasis are shown in G, while the respective data for potentially negative regulators are indicated in H. The *Tnfrsf17* (BCMA) sgRNA was depleted only in the TI sgRNA screen. ns, nonsignificant. Multiple replicate samples were analyzed in two independent sgRNA screening experiments in response to TD or TI immunization, as described in detail in Fig. S3 D (TD) and Fig. S4 D (TI). FC, fold change; PB, plasmablast.

To screen for genes important for the development of TD B cell responses, we immunized recipient mice with NP-KLH/alum. At day 7, donor-derived splenic cells, which were enriched by immunomagnetic depletion of recipient cells, were used for flow cytometric sorting of GC B cells (CD19^+^CD95^+^GL7^+^) and plasmablasts (TACI^+^CD138^+^) (Fig. S3, B and C; and Materials and methods). Sorted cells from five or six individual recipient mice were pooled to create replicate samples (Fig. S3 D). Changes of sgRNA abundance in plasmablasts and GC B cells were identified relative to the infected donor B cells (before transfer), which indicated that essential genes were efficiently selected against in contrast to members of the nonexpressed control olfactory receptor (*Olfr*) gene family, as expected (Fig. 4 A). Statistical analyses of the significant changes in sgRNA abundance identified multiple genes that were required for the development or survival of both cell types (Fig. 4 B and Table S3). As expected, the screen identified *Prdm1* and *Irf4* as positive regulators of plasmablast development, while *Irf4*, but not *Prdm1*, was also essential for the generation of GC B cells (Fig. 4, B and C). *Rexo2*, which was previously validated as a regulator of plasmablast development in an *in vitro* CRISPR/Cas9 screen (Pinter et al., 2022), was also essential *in vivo* for the development of plasmablasts and GC B cells (Fig. 4, B and C). Several hits of the *in vivo* screen were identified as positive regulators involved in the control of the homeostasis of the endoplasmic reticulum (ER) or protein secretion, such as *Ern1*, *Ssr2*, *Ssr4*, *Manf*, *Erlec1*, *Herpud1*, *P4hb*, *Kderl3*, *Arl5a*, *Ap3s1*, and *Unc13b* (Fig. 4 C). Moreover, the screens revealed a group of novel genes, including enzymes, adhesion molecules, transporters, and signaling proteins, which may be important for the initial B cell response, plasmablast development, and/or homeostasis (Fig. 4, D and E). Several of these genes appear to code for common positive regulators, as their inactivation affected the generation of both plasmablasts and GC B cells (Fig. 4 D). Moreover, other gene hits encoding potential positive regulators may act in a cell type–specific manner as their inactivation interfered with the formation of either plasmablasts or GC B cells (Fig. S2 C). Notably, several sgRNA hits appear to code for cell type–specific negative regulators as their gene inactivation resulted in an increase of either plasmablasts or GC B cells (Fig. 4 E and Fig. S2 C).

**Figure S4.**
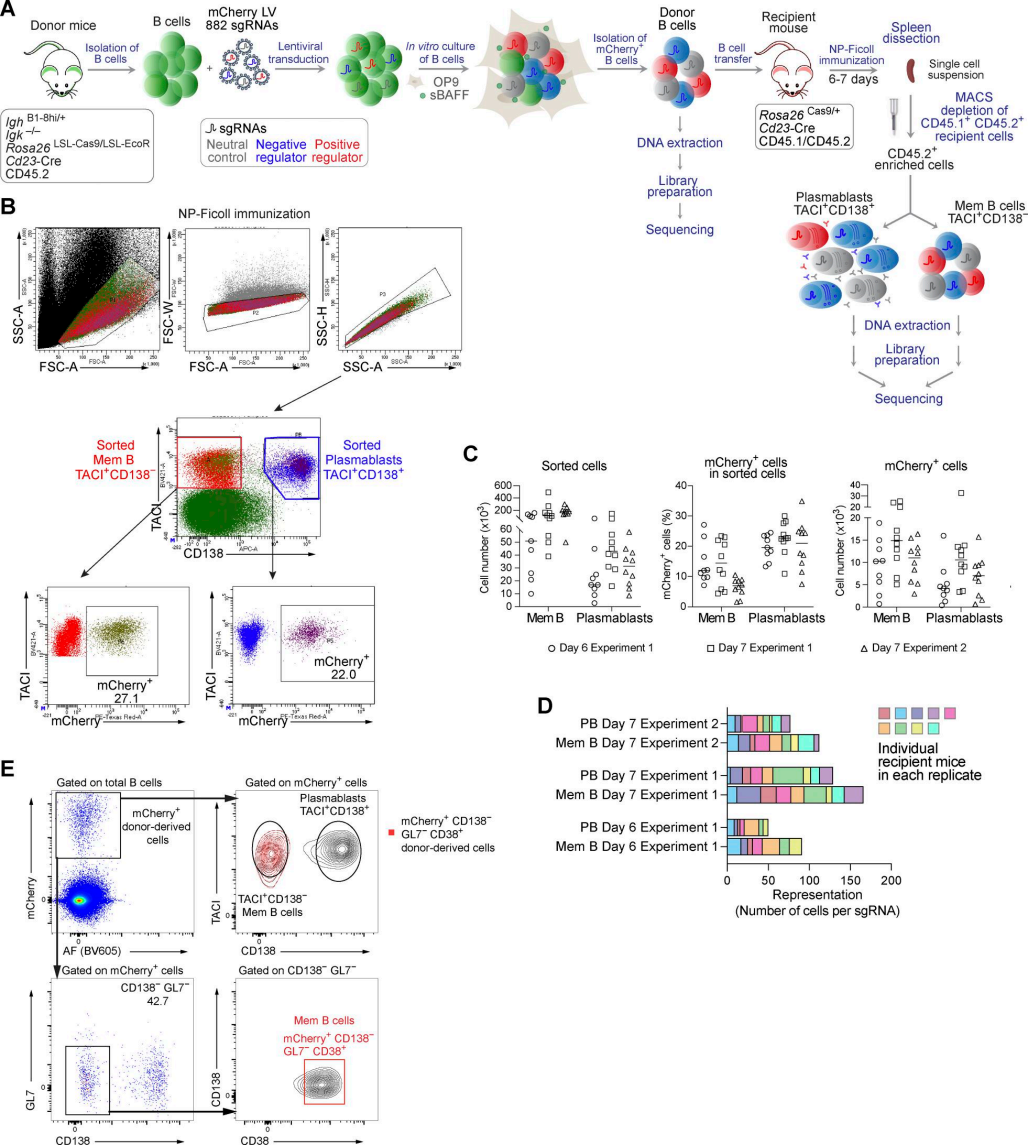
**CRISPR/Cas9 screen for identifying new regulators of the TI B cell responses. (A)** Schematic diagram of the *in vivo* CRISPR/Cas9 screening experiments used to study the TI B cell responses. The TI screening experiment was performed as described in detail for the TD screening experiment (Fig. S3 A), except that the transplanted mice were immunized with NP-Ficoll and that plasmablasts (TACI^+^CD138^+^) and memory B cells (Mem B, TACI^+^CD138^−^) were isolated at day 6 or 7 by flow cytometry. **(B)** Flow cytometric analysis of enriched mCherry^+^ CD45.2^+^ B cells from recipient mice. The gates used for the sorting of plasmablasts and memory B cells are indicated. The percentages of mCherry^+^ plasmablasts and mCherry^+^ memory B cells within the sorted cell population are shown. **(C)** Numbers of sorted plasmablasts and memory B cells (left), the percentage of mCherry^+^ plasmablasts and mCherry^+^ memory B cells within the sorted cell population (middle), and the numbers of sorted mCherry^+^ plasmablasts and mCherry^+^ memory B cells (right) were determined for individual recipient mice analyzed in two independent screening experiments. **(D)** Total sgRNA representation in each plasmablast and memory B cell replicate sample analyzed in two screening experiments. The sgRNA representation was estimated by dividing the number of mCherry^+^ plasmablasts or mCherry^+^ memory B cells by the number of the 882 sgRNAs constituting the sgRNA library. Each plasmablast and memory B cell replicate sample contained cells that were obtained from seven or nine recipient mice after pooling. Each colored box represents the individual contribution of each mouse to the sgRNA representation of the entire replicate sample. **(E)** Flow cytometric definition of mCherry^+^ memory B cells. Sorted mCherry^+^ donor B cells were transferred to recipient mice, which were immunized 16 h later with NP-Ficoll in PBS. Flow cytometric analysis of splenocytes 6 days after immunization is shown. Donor-derived mCherry^+^ cells were mainly TACI^+^CD138^+^ plasmablasts or CD138^−^GL7^−^CD38^+^TACI^+^ cells that phenotypically correspond to memory B cells.

To identify genes regulating the TI B cell responses, we immunized recipient mice after transfer of sgRNA library-transduced donor B cells with NP-Ficoll (Fig. S4 A). 6 or 7 days after immunization, donor-derived splenic cells were enriched and used for flow cytometric sorting of plasmablasts (TACI^+^CD138^+^) and TACI^+^CD138^–^ cells, which were enriched for memory B cells (mCherry^+^GL7^–^CD38^+^TACI^+^CD138^–^) (Fig. S4, B and E). Sorted cells from seven or nine individual recipient mice were pooled to create replicate samples (Fig. S4, C and D). By quantifying the changes in sgRNA abundance in plasmablasts and memory B cells compared with donor B cells, we identified multiple genes that potentially regulate the development or survival of both cell types (Fig. 4 F and Table S3). As many of the potential regulators found in the TI screens were also identified in the TD screens (Fig. 4, C–E), we show the respective data of these regulators for the TI screen in Fig. 4, G and H.

We next compared the regulators identified in our *in vivo* CRISPR/Cas9 screening experiments with the published hit list of the *in vitro* CRISPR/Cas9 screens that were performed with *in vitro*–generated plasmablasts (Newman and Tolar, 2021; Pinter et al., 2022; Trezise et al., 2023; Turner et al., 2022; Xiong et al., 2023; Chu et al., 2016). This comparison revealed that the majority (over 80%) of regulators identified in our study (Fig. 4, C–H; indicated in green color) were uniquely identified in the *in vivo* screen. In summary, our *in vivo* CRISPR/Cas9 screening system identified novel regulators involved in the control of TD and TI B cell responses, plasmablast formation, and/or homeostasis.

### Validation of potential regulators of B cell responses

**Figure 5.**
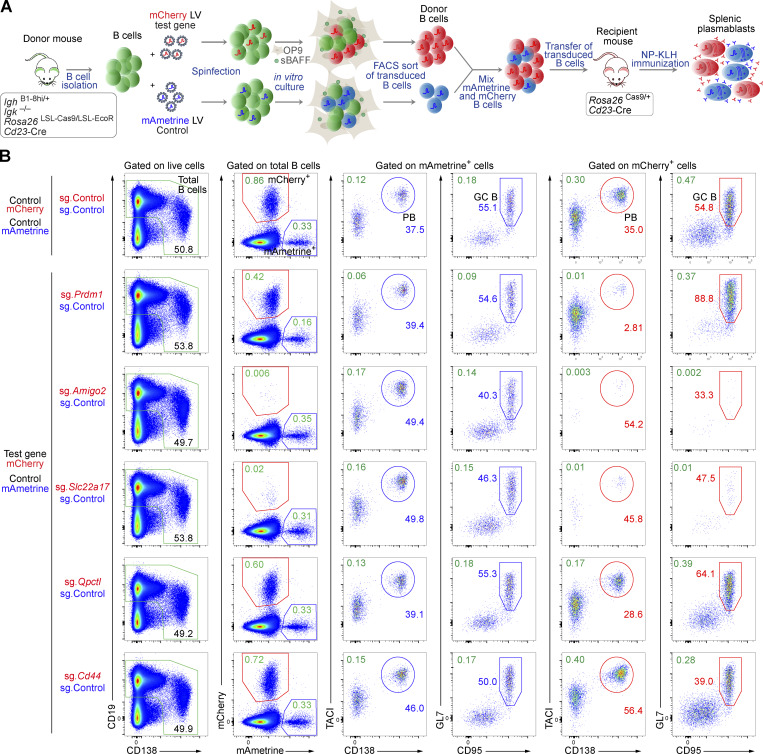
**Validation of gene hits of the TD B cell responses by sgRNA-mediated gene inactivation. (A)** Schematic diagram of the experiments used for validating potential regulators identified by the *in vivo* CRISPR/Cas9 screens. Naïve splenic B cells of the donor genotype were transduced with LV particles expressing the mCherry reporter protein and a sgRNA targeting the gene of interest. In parallel, donor B cells were transduced with LV particles expressing the mAmetrine reporter protein and the control sgRNA (sg.*Chr1*). After infection, the cells were cultured *in vitro* for 3 days in the presence of OP9 cells and sBAFF. Transduced mAmetrine^+^ and mCherry^+^ B cells were sorted by flow cytometry, mixed, and transferred to recipient mice, which were then immunized with NP-KLH/alum 16 h later. The frequency of mAmetrine^+^ and mCherry^+^ PBs in the spleen was analyzed by flow cytometry 7 days after immunization. **(B)** mCherry^+^ and mAmetrine^+^ sgRNA-expressing B cells were mixed at a 2.5:1 ratio and transferred into recipient mice, followed by immunization. Flow cytometric analysis of splenocytes from recipient mice was performed 7 days after immunization. mCherry^+^ B cells expressed either the control sgRNA (sg.*Chr1*) or sgRNAs targeting *Prdm1* (sg.*Prdm1*), positive regulator genes (sg.*Amigo2*, sg.*Slc22a17*, and sg.*Qpctl*), and a negative regulator gene (sg.*Cd44*). The percentage of mCherry^+^ (red gate) and mAmetrine^+^ (blue gate) B cells among the total B cells (CD19^+^CD138^–/lo^ and CD19^lo^CD138^+^; green gate) is shown. The percentages of mCherry^+^ and mAmetrine^+^ PBs (TACI^+^CD138^+^) and GC B cells (GL7^+^CD95^+^) in total B cells (green numbers) and within the mCherry^+^ B cells (red numbers) or mAmetrine^+^ B cells (blue numbers) are indicated. PB, plasmablast.

**Figure 6.**
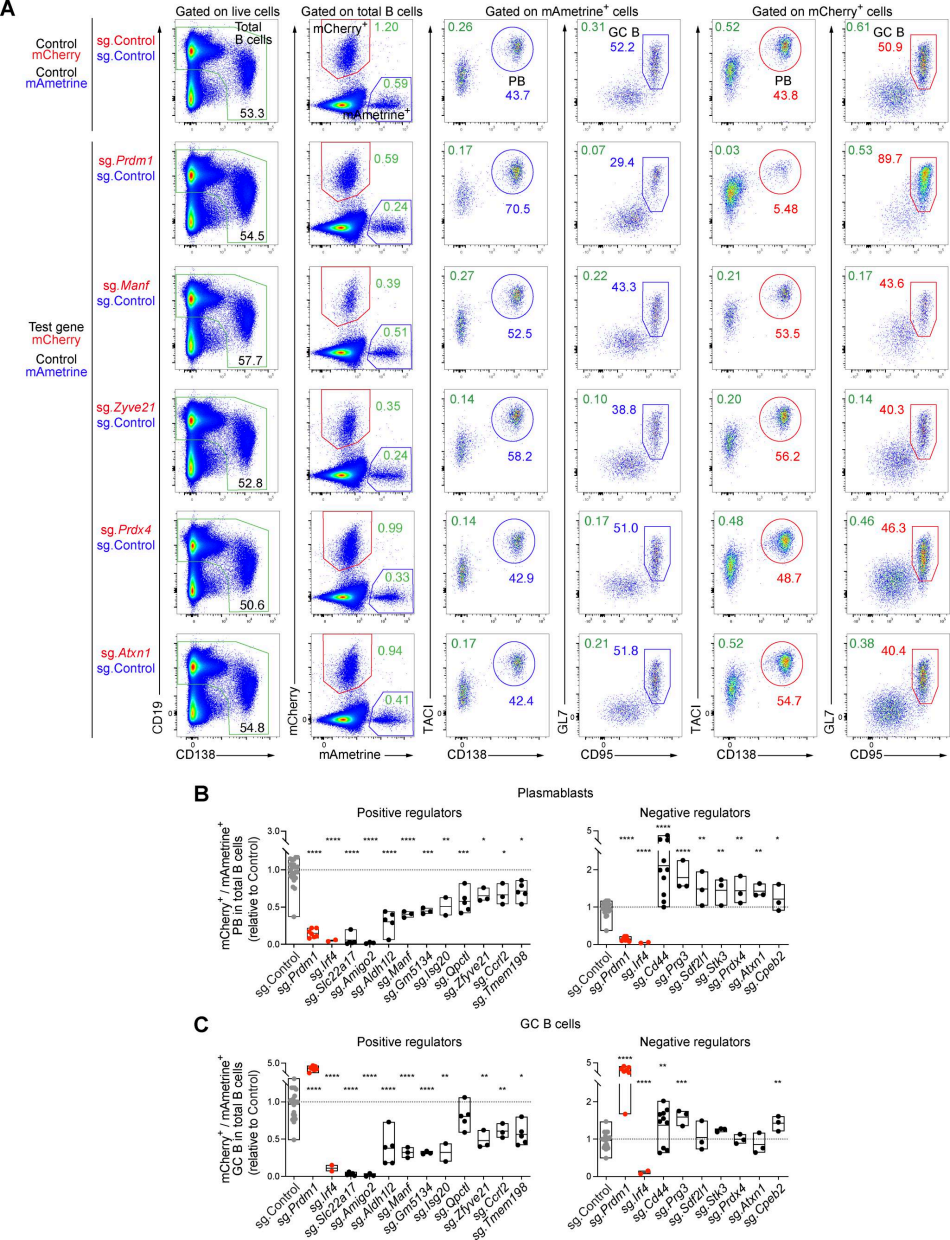
**Validation of positive and negative regulators of plasmablast and GC B cell formation by sgRNA-mediated gene inactivation. (A)** mCherry^+^ and mAmetrine^+^ sgRNA-expressing B cells were mixed at a 2.5:1 ratio and transferred into recipient mice, followed by NP-KLH immunization. Flow cytometric analysis of splenocytes from recipient mice was performed 7 days after immunization. mCherry^+^ B cells expressed either the control sgRNA (sg.*Chr1*) or sgRNAs targeting *Prdm1* (sg.*Prdm1*), positive regulator genes (sg.*Manf* and *sgZyve21*), or negative regulator genes (sg.*Prdx4* and sg.*Atxn1*). The percentage of mCherry^+^ (red gate) and mAmetrine^+^ (blue gate) B cells among the total B cells (CD19^+^CD138^–/lo^ and CD19^lo^CD138^+^; green gate) is shown. The percentages of mCherry^+^ and mAmetrine^+^ plasmablasts (TACI^+^CD138^+^) and GC B cells (GL7^+^CD95^+^) in total B cells (green numbers) and within the mCherry^+^ B cells (red numbers) or in mAmetrine^+^ B cells (blue numbers) are indicated. **(B and C)** Analysis of the loss (left) or gain (right) of plasmablasts and GC B cells upon sgRNA-mediated inactivation of candidate genes coding for positive or negative regulators, respectively. The ratio of the percentage of mCherry^+^ plasmablasts (B) or mCherry^+^ GC B cells (C) (expressing the sg.Control [sg.*Chr1*] or sgRNAs targeting the indicated genes) versus mAmetrine^+^ plasmablasts (B) or mAmetrine^+^ GC B cells (C) (expressing the sg.Control) was determined in total B cells, respectively. The calculation of the different ratios is explained in detail in Fig. S5 C. The ratios, which were determined by analyzing different control mice (gray), experimental mice (black), and mice with inactivation of the known regulator genes *Prdm1* or *Irf4* (red), are indicated. Statistical data are shown as mean values with SEM and were analyzed by the multiple unpaired *t* test with Holm–Šídák’s multiple comparisons test; *P < 0.05; **P < 0.01; ***P < 0.001; ****P < 0.0001. Each dot represents one mouse. The validation experiments were performed at least two times.

**Figure S5.**
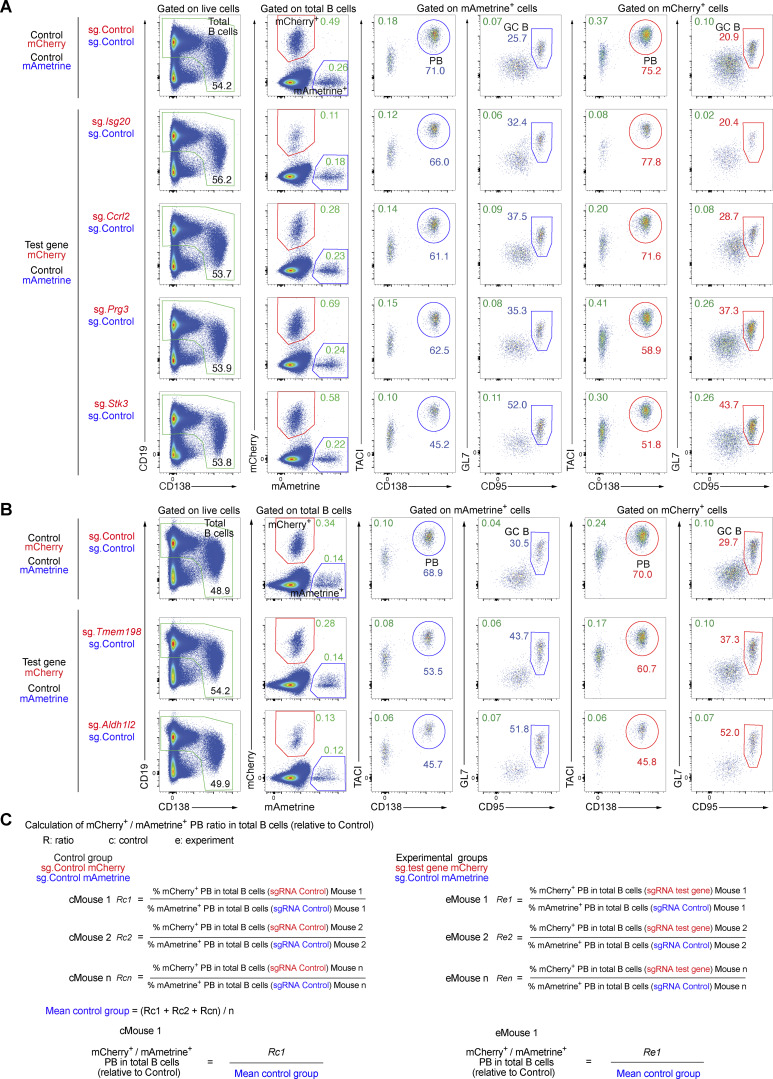
**Validation of the indicated regulators of TD B cell responses by sgRNA-mediated gene inactivation. (A and B)** Flow cytometric analysis of two independent validation experiments performed by sgRNA-mediated inactivation of putative positive and negative regulators. The outline of the strategy and flow cytometric analysis of the validation experiments is explained in detail in Fig. 5 A. mCherry^+^ and mAmetrine^+^ sgRNA-transduced B cells were mixed at a ratio of 2.5:1 and transferred into recipient mice, followed by immunization with NP-KLH/alum. Flow cytometric analysis of splenocytes from recipient mice was performed 7 days after immunization. mCherry^+^ donor B cells expressed the control sgRNA (sg.*Chr1*) or sgRNAs targeting positive regulator genes (sg.*Isg20*, sg.*Ccrl2*, sg.*Tmem198*, and sg.*Aldh1l2*) or negative regulator genes (sg.*Prg3* and sg.*Stk3*), while the mAmetrine^+^ donor B cells expressed only the control sgRNA. The percentages of mCherry^+^ and mAmetrine^+^ plasmablasts (TACI^+^CD138^+^) and GC B cells (GL7^+^CD95^+^) in total B cells (CD19^+^CD138^–/lo^ and CD19^lo^CD138^+^; green), in mCherry^+^ cells (red), or in mAmetrine^+^ cells (blue) are shown. The ratios of mCherry^+^ versus mAmetrine^+^ plasmablasts and GC B cells were quantified and calculated, as explained in C, and are shown in Fig. 6, B and C. **(C)** Calculations of the ratio of mCherry^+^ to mAmetrine^+^ plasmablasts in total B cells relative to the control. The corresponding results are shown in Fig. 6 B. First, the ratio (R) of the percentage of mCherry^+^ plasmablasts (sg.Control [sg.*Chr1*]) in total B cells versus the percentage of mAmetrine^+^ plasmablasts (sg.Control) in total B cells was determined for each control (c) mouse. Subsequently, the mean value of the control group (mean control group) was determined by dividing the sum of all Rc values by the total number of control mice analyzed. In parallel, the ratio (R) of the percentage of mCherry^+^ plasmablasts (for each sgRNA test gene) in total B cells versus the percentage of mAmetrine^+^ plasmablasts (sg.Control) in total B cells was determined for each experimental (e) mouse. We then normalized the Re value of each experimental mouse by dividing it by the mean value of the control group, which allowed to plot all Re values of the different mice in the same graph by setting the mean Rc value to 1 as shown in Fig. 6 B. The same calculation was used to determine the ratios for GC B cells (Fig. 6 C).

Several of the genes identified in the *in vivo* screens are potentially interesting as their role in regulating late B cell responses is so far unknown. We selected 15 potentially positive and 9 potentially negative regulators for validation in a competitive experimental setting (Fig. 5 A). Briefly, recipient mice were injected with a mixture of mCherry^+^ and mAmetrine^+^ donor B cells at a ratio of ∼2.5 to 1. The mCherry^+^ cells expressed a sgRNA targeting a gene of interest, while the mAmetrine^+^ cells expressing the control sgRNA (sg.*Chr1*) were used as reference cell population. The proportion of mCherry^+^ versus mAmetrine^+^ cells in recipient mice was analyzed 7 days after NP-KLH/alum immunization. Control mice that received sg.Control-expressing mCherry^+^ and sg.Control-expressing mAmetrine^+^ cells had similar frequencies of mCherry^+^ and mAmetrine^+^ plasmablasts or GC B cells (Fig. 5 B). In contrast, mice that received a mixture of sg.*Prdm1*-expressing mCherry^+^ and sg.Control-expressing mAmetrine^+^ cells lacked mCherry^+^ plasmablasts (Fig. 5 B and Fig. 6 A). Inactivation of *Amigo2* and *Slc22a17*, encoding two potentially positive regulators, led to an almost complete absence of mCherry^+^ cells, suggesting that both genes may play an essential function at early stages of B cell activation. Instead, the inactivation of *Cd44*, encoding a potentially negative regulator, resulted in the accumulation of mCherry^+^ plasmablasts (Fig. 5 B). As we performed several validation experiments for the different genes (Fig. 5 B; Fig. 6 A; and Fig. S5, A and B), we calculated the normalized mCherry^+^/mAmetrine^+^ cell ratio for each gene in plasmablasts and GC B cells, as explained in Fig. S5 C, and show the validation results for all genes tested in Fig. 6, B and C.

Inactivation of other positive regulator genes, such as *Aldh1l2*, *Manf*, *Gm5134*, *Isg20*, *Zfyve21*, *Tmem198*, and *Ccrl2*, reduced the proportion of mCherry^+^ plasmablasts and GC B cells relative to the respective mAmetrine^+^ control cells (Fig. 6, B and C). *Qpctl* inactivation resulted in a preferential reduction of mCherry^+^ plasmablasts. In contrast, the inactivation of potentially negative regulator genes, such as *Prg3*, *Sdf2l1*, *Stk3*, *Prdx4*, *Atxn1*, and *Cpeb2*, caused an accumulation of mCherry^+^ plasmablasts and in some cases also an increase in GC B cells relative to the reference control mAmetrine^+^ cells (Fig. 6, B and C), consistent with the initial screening result (Fig. 4 E). In summary, these validation experiments confirmed that many of the potential regulators identified in our *in vivo* screens seem to play a critical role in the process of B cell activation, GC B cell formation, plasmablast differentiation, or survival.

## Discussion

B cell–mediated immune responses are crucial for immunity and long-term protection against pathogens, but are also involved in various diseases such as autoimmunity, allergy, and cancer (Cyster and Allen, 2019; Nutt et al., 2015). Here, we developed an *in vivo* screening method for pooled sgRNA CRISPR/Cas9 screens to identify new regulators of B cell responses. This model system relies on the *in vivo* activation of sgRNA-expressing naïve donor B cells and the differentiation of these cells into effector B cells within the microenvironment of secondary lymphoid organs. To achieve this, we generated a mouse, in which mature B cells ectopically express the receptor SLC7A1 for the ecotropic envelope protein of the murine leukemia virus, which allows efficient *ex vivo* transduction of naïve B cells without activation. These transduced naïve B cells were cultured *in vitro* for 3 days on OP9 cells with sBAFF, which preserved their naïve phenotype and cell viability, before they were sorted and transferred to recipient mice. Upon immunization with model antigens, these sgRNA-carrying naïve donor B cells could mount TD and TI B cell immune responses in the recipient mice. Finally, using this novel system, we performed several *in vivo* CRISPR/Cas9 screens, which identified novel genes important for the development of B cell responses *in vivo*.

One of the main challenges in developing the *in vivo* model system was the transduction of naïve B cells. Mouse B cells are commonly transduced by LVs after activation via Toll-like receptor stimulation, such as TLR9 with CpG, TLR4 with LPS, or CD40 receptor stimulation in conjunction with IL-4 (Janssens et al., 2003; Rossi et al., 2003). However, we considered it essential for a successful screen that B cells should be activated *in situ* within the microenvironment of secondary lymphoid organs. Mature naïve B cells express only low levels of the transporter SLC7A1 (EcoR), the receptor for the ecotropic envelope protein of the murine leukemia virus. Upon B cell activation, the expression of SLC7A1 is rapidly increased, leading to efficient LV infection. Therefore, we adopted the strategy of expressing the receptor at higher levels in naïve mature B cells by generating the *Rosa26*^LSL-EcoR^ allele and crossing it into *Cd23*-Cre mice to induce specific expression only at late B cell stages. As SLC7A1 is a cationic L-amino acid transporter (Closs et al., 2006), its overexpression could potentially affect the function of mature B cells. At steady state and upon immunization, B cell subpopulations and antibody-secreting cells were, however, present at normal frequencies in the spleen of *Cd23*-Cre *Rosa26*^LSL-EcoR/+^ mice compared with control mice, demonstrating that mature B cells and plasmablasts are not affected by higher expression of SLC7A1.

One important limitation of our study was the number of genes that could be tested simultaneously by the *in vivo* CRISPR/Cas9 screening system. A minimal representation of ∼500 cells per sgRNA is usually recommended at the end of the screening experiment (Doench, 2018; Bock et al., 2022). By using a library size of 882 sgRNAs, we achieved this sgRNA representation for plasmablasts and GC B cells at day 7 of the TD screening experiments (Fig. S2 D and Fig. S3 D). It has been reported that a relatively small fraction of the intravenously injected B cells survive and engraft *in vivo* in secondary lymphoid organs (Taylor et al., 2015). Our direct flow cytometric measurements identified 13,400 or 21,600 infected mCherry^+^ B cells in the spleen of one mouse at day 3 after the initial transfer of 200,000 donor B cells in the TD or TI immunization experiment, respectively (Fig. 3 B, Fig. S1 D, and Fig. S2 D). Under the assumption that each cell was infected by only one sgRNA-expressing LV of the screening library consisting of 882 sgRNAs, the measured mCherry^+^ B cell numbers resulted in a representation of 15 or 25 B cells per sgRNA for one mouse at day 3 after TD or TI immunization, respectively (Fig. S2 D). As the sorted B cells from five or nine mice were pooled to generate one replicate sample (Fig. S3 D and Fig. S4 D), this led to a reasonably high representation of 75 or 225 cells per sgRNA for each replicate sample at the bottleneck stage (day 3) of the TD or TI screening experiment (Fig. S2 D). Notably, the sgRNA representation at day 7 was strongly increased in plasmablasts (524 cells/sgRNA) and GC B cells (664/sgRNA) at the end of the TD experiment, which testifies to the high quality of this screening experiment. In contrast, the sgRNA representation was lower in plasmablasts (90 cells/sgRNA) and memory B cells (103 cells/sgRNA) at day 7 of the TI experiment, which is likely caused by the lower cell proliferation observed upon TI immunization. Although the lower sgRNA representation somehow limited the relevance of the TI screening experiments, we found nevertheless many common positive and negative regulators by analyzing plasmablasts generated by TD and TI immunization.

Employing the *in vivo* system, we identified 48 genes that are potentially important for the development of antibody-secreting cells, and thus for B cell–mediated immune responses. These genes include well-known positive regulators such as the transcription factors *Irf4* (Sciammas et al., 2006; Klein et al., 2006) and *Prdm1* (Tellier et al., 2016; Minnich et al., 2016). Plasma cells produce large amounts of antibodies that require expansion of their ER (Nutt et al., 2015; Bettigole and Glimcher, 2015). It was expected that several positive regulators found in the CRISPR/Cas9 screens are involved in the control of the homeostasis of the ER, many of which were identified in previous *in vitro* CRISPR/Cas9 screening studies (Pinter et al., 2022; Trezise et al., 2023; Xiong et al., 2023). The screening experiments performed here also identified a number of genes encoding regulators of plasma cell development and/or survival that were not found in previously performed *in vitro* CRISPR/Cas9 screens. These novel genes are most likely relevant for the *in vivo* regulation of B cell responses. We used the *in vivo* model system in a competitive setting to validate 15 positive and 9 negative regulators of these unique hits, which indicated that 70% (17) of these genes could be validated. Several of the validated genes appear to regulate the development of both plasmablasts and GC B cells. This finding suggests that these genes may play a role in early B cell activation preceding the commitment to the GC B cell and plasma cell lineages. Other genes were specifically implicated in the formation of plasmablasts or GC B cells, respectively. Ultimately, gene-specific deletion in mature B cells will be required to investigate the precise function of these novel genes *in vivo* in the mouse.

*Amigo2* and *Slc22a17* have emerged as prominent genes among the validated positive regulators. Both TD and TI screening experiments identified Amigo2 and Slc22a17 as essential positive regulators of the development or survival of plasmablasts, GC B cells, and memory B cells. Amigo2 is an adhesion molecule that belongs to the immunoglobulin and leucine-rich repeat protein superfamily, which was primarily studied in the nervous system (Kuja-Panula et al., 2003; Ono et al., 2003). It has been shown to be a neuronal activity–dependent gene with an important role in signal transduction by inhibiting apoptosis, and thus inducing neuronal cell survival (Ono et al., 2003). Moreover, Slc22a17 has been implicated in iron transport by binding and internalizing iron-loaded lipocalin-2, which promotes cell survival by increasing the intracellular iron concentration. In contrast, iron-lacking lipocalin-2, once imported by Slc22a17, binds and exports intracellular iron, which results in Bim1-mediated apoptosis (Devireddy et al., 2005; Yang et al., 2002). Hence, as both Amigo2 and Slc22a17 appear to control cell survival, their loss likely interferes with the B cell immune response at an early stage.

*Qpctl* encodes a glutaminyl-peptide cyclotransferase–like protein. QPCTL is a Golgi-resident enzyme that catalyzes the cyclization of N-terminal glutamine and glutamic acid residues to a pyroglutamate residue on target proteins (Stephan et al., 2009). *Qpctl* was identified in a previous *in vitro* CRISPR/Cas9 screen as a regulator of antibody secretion (Trezise et al., 2023). In our TD screening experiments, *Qpctl* inactivation led to the depletion of plasmablasts without affecting GC B cells, which was supported by validation experiments. However, in the TI screening experiments, inactivation of the *Qpctl* gene affected the development of both plasmablasts and memory B cells. QPCTL was recently shown to modify the monocyte chemoattractants CCL2 and CCL7, which protects them from proteolytic inactivation, thus influencing monocyte migration and homeostasis (Barreira da Silva et al., 2022). What the relevant target proteins of QPCTL may be in B cells is still unknown and may thus be an interesting question to be addressed by future investigation.

*Zfyve21* and *Isg20* were previously described to regulate antibody secretion by plasma cells (Trezise et al., 2023). In addition, our TD screen now demonstrated that upon inactivation of either gene, the development or survival of plasmablasts and GC B cells was impaired. ZFYVE21 is a member of a protein family that shares the FYVE domain for binding phosphatidylinositol-3-phosphate in the plasma membrane and that can modulate cell adhesion and migration by regulating focal adhesion (Nagano et al., 2010). In endothelial cells, ZFYVE21 promotes the degradation of endosome-associated PTEN, leading to increased PI(3,4,5)P3 levels that facilitate AKT-dependent recruitment of the NF-κB–inducing kinase (Fang et al., 2019). On the other hand, ISG20 is a type I interferon–induced protein belonging to the DEDD 3′-5′ exonuclease superfamily. It exhibits RNA exonuclease activity, which has been associated with the inhibition of a broad range of RNA viruses. How these two proteins, ZFYVE21 and ISG20, regulate B cell responses remains to be elucidated.

One of the most prominent genes, acting as a negative regulator of B cell responses, is *Cd44*, which encodes a transmembrane glycoprotein involved in cell adhesion and signal transduction (Ponta et al., 2003)*. Cd44* was previously shown to be dispensable for B lymphopoiesis (Bradl et al., 2004), although this study did not examine plasma cell frequencies *in vivo*. *In vitro* experiments have shown that the CD44 ligand hyaluronic acid can induce B cell activation, proliferation, and differentiation (Rafi et al., 1997). CD44 engagement with anti-CD44 monoclonal antibodies promotes *in vitro* survival of isolated bone marrow plasma cells (Cassese et al., 2003). CD44 has furthermore been implicated in lymphocyte recirculation (Protin et al., 1999). Our TD and TI screening results revealed a specific increase of splenic plasmablasts that were generated from sg.*Cd44*-expressing B cells. Furthermore, validation experiments confirmed a preferential increase of plasmablasts upon *Cd44* inactivation in B cells. Future studies involving specific inactivation of *Cd44* in mature B cells will shed light on the two interesting hypotheses of whether *Cd44*-deficient plasmablasts accumulate in the spleen due to impaired migration and egress from the organ or due to reduced apoptosis leading to increased survival.

The identified negative regulator gene *Atxn1* codes for a polyglutamine protein that is localized in the nucleus and interacts with the transcriptional repressor Capicua to regulate gene transcription (Lam et al., 2006). In a multiple sclerosis (MS) mouse model, Atxn1 was recently found to repress B cell activation and cytokine production. Furthermore, *Atxn1* KO mice have increased serum levels of IgG and IgM antibodies (Didonna et al., 2020). Although these results are consistent with our identification of Atxn1 as a negative regulator of plasmablast generation, the differentiation of *Atxn1*-deficient B cells into plasma cells has not yet been studied.

Other validated negative regulator genes that were newly found in our screens include *Stk3*, *Sdf2l1,* and *Prdx4*. The serine/threonine kinase 3 (Stk3) is a key component of the Hippo signaling pathway, which regulates immune cell function by regulating cellular adhesion, intracellular signaling, metabolism, cell growth, and survival (Hong et al., 2018; Ardestani et al., 2018). The analysis of the Hippo signaling pathway in the B cell lineage has mainly focused on the homolog Stk4 (Bai et al., 2016; Abdollahpour et al., 2012), and, to date, no study has clearly shown an effect of this pathway on the generation, survival, or migration of plasma cells. Stromal cell–derived factor 2 like 1 (SDF2L1) is an ER-localized protein, whose levels are increased in response to ER stress (Fukuda et al., 2001; Sasako et al., 2019). In pancreatic β-cells, SDF2L1 plays a role in ER-associated degradation of misfolded proinsulin (Fukuda et al., 2001). Peroxiredoxin-4 (PRDX4) is a 2-cysteine peroxiredoxin that is found predominantly in the ER (Rhee et al., 2012; Tavender et al., 2008). It is a major component of the ER oxidative protein-folding pathway and acts as a sensor of H_2_O_2_ in protein disulfide isomerase–mediated protein folding (Rhee et al., 2012; Sato et al., 2013; Yan et al., 2015). How the two ER proteins SDF2L1 and PRDX4, which regulate plasma cell homeostasis, lead to the accumulation of plasma cells in the spleen upon immunization remains to be elucidated.

Multiple studies have used *in vivo* CRISPR/Cas9 screens to identify new regulators of different T cell–related processes by using focused pooled sgRNA libraries targeting genes involved in a particular pathway (Huang et al., 2021; Fu et al., 2021; Huang et al., 2022). In these studies, T cells were activated *in vitro* prior to transduction with sgRNA-carrying viral particles. Our approach based on ectopic SLC7A1 expression may also be useful for studying T cell differentiation by allowing the transduction of naïve T cells *ex vivo* followed by their proper activation *in vivo* upon antigen encounter within secondary lymphoid organs. This approach could allow for the screening of regulators implicated in the homeostasis or activation of naïve T cells.

Lastly, our *in vivo* model system may also be suitable for investigating the importance of genes in specific pathways during the development of B cell–mediated immune responses. For instance, it could be used to explore the role of individual genes or groups of genes in the development of a particular effector B cell subset, such as light or dark zone GC B cells, memory B cells, or plasmablasts expressing a specific class of immunoglobulin.

## Materials and methods

### Mice

All mice were maintained on the C57BL/6J genetic background: *Rosa26*^Cas9/+^ (Platt et al., 2014), *Rosa26*^LSL-Cas9/+^ (Platt et al., 2014), *Rosa2*6^LSL-miR17-92/LSL-miR17-92^ (Xiao et al., 2008), *Igh*^B1-8hi/+^ (Shih et al., 2002), *Igk*^–/–^ (Zou et al., 1993), and transgenic *Cd23*-Cre (Kwon et al., 2008) mice. Experimental and control mice were cohoused under standard pathogen–free conditions at a temperature of 22°C and 55% humidity, with a day cycle of 14-h light and 10-h dark and with unrestricted access to food and water. Mice were euthanized by carbon dioxide inhalation. All experiments were performed with mice at the age of 8–14 wk and according to valid project licenses approved and regularly controlled by the Austrian Veterinary Authorities.

### Generation of the *Rosa26*^LSL-EcoR^ allele

To create the *Rosa26*^LSL-EcoR^ allele, *Slc7a1* cDNA was first cloned into the CAG-STOP-eGFP-Rosa26 CTV plasmid (Addgene plasmid no. 15912) by replacing the IRES-eGFP sequence with *Slc7a1* cDNA to generate the CAG-STOP-Slc7a1-Rosa26 plasmid. A 3,564-bp-long DNA fragment was PCR-amplified from the CAG-STOP-Slc7a1-Rosa26 plasmid using upstream (5′-CTG​GCA​CTT​CTT​GGT​TTT​CC-3′) and downstream (5′-GCT​GCA​TAA​AAC​CCC​AGA​TG-3′) primers. The *Rosa26*^LSL-EcoR^ allele was generated by CRISPR/Cas9-mediated genome editing in mouse two-cell embryos (2C-HR-CRISPR) (Gu et al., 2018). For this, two-cell embryos of the *Rosa2*6^LSL-miR17-92/LSL-miR17-92^ genotype (on the C57BL/6J background) were injected with Cas9 protein, three appropriate sgRNAs (linked to the scaffold tracrRNA; Table S1), and the double-stranded 3,564-bp DNA repair template to generate the *Rosa26*^LSL-EcoR^ allele (Fig. 2 A). Cas9 protein and backbone-modified sgRNAs were obtained from Integrated DNA Technologies (IDT). Correct targeting of the *Rosa26*^LSL-EcoR^ allele was verified by DNA sequencing of the respective PCR fragments. The *Rosa26*^LSL-EcoR^ allele was genotyped by amplification of a 692-bp PCR fragment with the primers 5′-TTA​AGC​CTG​CCC​AGA​AGA​CT-3′ and 5′-TGA​CAG​GGT​CAG​TCC​TCC​TC-3′. In a separate PCR, the wild-type *Rosa26* allele was genotyped by amplification of a 170-bp PCR fragment with the primers 5′-CTC​TTC​CCT​CGT​GAT​CTG​CAA​CTC​C-3′ and 5′-TCC​CGA​CAA​AAC​CGA​AAA​T-3′.

### Antibodies

The following monoclonal antibodies (clone, fluorophore, catalog number, manufacturer) were used for flow cytometric analysis of cells: CD19 (1D3, BV786, 563333; BD Biosciences), CD21/CD35 (7G6, BV605, 747763; BD Biosciences), CD23 (B3B4, PE/Cyanine7, 101614; BioLegend), CD38 (90, PE, 102718; BioLegend), B220/CD45R (RA3-6B2, BV510, 103248; PE, 103208; BioLegend), CD45.1 (A20, Biotin, 110704; BioLegend), CD69 (PE/Cyanine7, 104512, H1.2F3; BioLegend), CD93 (AA4.1, PE, 136504; BioLegend), CD95/Fas (Jo2, PE/Cyanine7, 557653; BD Biosciences), CD138 (281-2, APC, 142506; BV605, 142516; BioLegend), CD267/TACI (8F10, PE, 133404; BioLegend; BV421, 742840; BD Biosciences), IgD (11.26c, BV421, 405725; BioLegend; 11.26c, FITC, 405703; BioLegend), IgM (II/41, PerCP-eFluor 710, 46-5790-82; Thermo Fisher Scientific), GL7 (GL-7, PerCP/Cyanine5, 144610; BioLegend), and SLC7A1 (SA191A10, PE, 150504; BioLegend) antibodies.

### Definition of cell types by flow cytometry

The different hematopoietic cell types were identified by flow cytometry using an LSRFortessa flow cytometer (BD Biosciences) or sorted by using FACSAria II and FACSAria III cell sorters operated using BD FACSDiva software (version 8.0) as follows: immature B (B220^+^CD19^+^CD93^+^), T1 (B220^+^CD19^+^CD93^+^IgM^+^CD23^–^), T2 (B220^+^CD19^+^CD93^+^ IgM^+^CD23^+^), mature B (B220^+^CD19^+^CD93^–^), MZ B (B220^+^CD19^+^CD93^–^CD21^hi^CD23^lo/–^), FO B (B220^+^CD19^+^CD93^–^CD21^int^CD23^hi^), GC B (B220^+^CD19^+^CD95^+^GL7^+^), plasmablasts (CD138^+^TACI^+^), and total B cells (CD19^+^B220^+^). For analysis of the validation experiments, total splenic B cells were defined as the sum of CD19^+^ and CD138^+^ cells.

### *In vitro* B cell cultures

CD43^–^ B cells were enriched from the spleen or lymph nodes of mice by immunomagnetic depletion of non-B cells using CD43 (Ly-48) MicroBeads (Miltenyi Biotec). B cells were cultured in B cell medium (RPMI 1640; Gibco) supplemented with 25 mM HEPES, 10% heat-inactivated FBS (Gibco), 1 mM glutamine (Gibco), penicillin/streptomycin (Gibco), and 50 μM β-mercaptoethanol (Gibco). The cells were seeded at a density of 2 × 10^6^ cells in 2 ml of medium. We tested different *in vitro* conditions to culture naïve B cells, preserving their viability and naïve state. In the reference control cultures, B cells were kept only in B cell medium or LPS from *Escherichia coli* (L4130; Sigma-Aldrich) was added to the cultures at a concentration of 25 μg/ml. Additionally, B cells were cultured with 10 ng/ml human recombinant sBAFF (60-mer, AG-40B-0112; AdipoGen Life Sciences) or on stromal OP9 cells in combination with or without sBAFF. OP9 cells were added at a density of 1.5–2 × 10^5^ cells in 2 ml of medium. The cultures were maintained for 3 days. The cells were stained with the Viability Dye eFluor 780 (Thermo Fisher Scientific) to analyze cell viability and with different B cell surface marker–specific antibodies for phenotypic characterization by flow cytometry. In some experiments, CD43^–^ B cells were labeled with 5 μM CellTrace Violet dye (Thermo Fisher Scientific) in PBS for 20 min at 37°C. After washing with B cell medium, the cells were cultured under different conditions for 3 days. Cell Trace Violet dye dilution was used as a readout of cell proliferation.

### *In vitro* plasmablast differentiation

Plasmablasts were generated *in vitro* by stimulation of CD43^–^ B cells with 0.2 μM CpG (ODN 1826; InvivoGen) plus 10 ng/ml IL-4 (404-ML-025/CF; R&D Systems) and 10 ng/ml IL-5 (405-ML-025; R&D Systems). CD43^–^ B cells were plated at a density of 1 × 10^6^ cells per 2 ml of B cell medium. After 4 days, the cultures were analyzed using flow cytometry to determine the percentage of plasmablasts (CD138^+^CD19^lo^).

### Immunizations

To induce a TD NP-specific response, mice were immunized intraperitoneally with 100 µg NP-KLH (IMMB1-042; Biosearch Technologies) in alum adjuvant. To induce a TI NP-specific immune response, mice received an intraperitoneal injection of 50 μg of NP-conjugated high-molecular-weight polysaccharide Ficoll (NP-Ficoll, IMMA1-008; Biosearch Technologies) in PBS.

### Immunohistological analysis

Mouse spleens were dissected and fixed with 4% paraformaldehyde in PBS for 1 h, cryopreserved with 30% sucrose in PBS overnight, and embedded in Tissue-Tek O.C.T. Compound (Sakura). For immunofluorescence staining, 10-μm cryosections were fixed with cold acetone, hydrated in PBS, and blocked with 2 μg/ml anti-mouse CD16/32 (2.4G2, 553142; BD Biosciences) antibody diluted in 5% BSA in PBS for 30 min. Sections were stained for 2 h with Alexa Fluor 488–conjugated anti-mouse IgD (11-26c.2a, 405718; BioLegend), Alexa Fluor 647 anti-mouse IRF4 (IRF4.3F4, 646408; BioLegend), rabbit polyclonal anti-mCherry (ab167453; Abcam), and BV421 anti-mouse TCRβ (H57-597; BioLegend) antibodies, diluted with a solution containing 2 μg/ml anti-mouse CD16/32, 1% BSA in PBS. Finally, the sections were incubated with donkey anti-rabbit IgG (H+L) Alexa Fluor 568 (A10042; Invitrogen) diluted in PBS containing 2 μg/ml anti-mouse CD16/32 and 1% BSA and mounted with ProLong Diamond Antifade Mountant (Invitrogen). The sections were imaged using a Zeiss LSM 880 confocal microscope operated by ZEN Black (version 2.3, Zeiss) software and a 20×/0.8 Plan-Apochromat lens at zoom factor 1 (415 nm/pixel) (Zeiss). Images were acquired with identical settings for the laser power, detector gain, and amplifier offset, with pinhole diameters set for one airy unit. The tile regions were stitched, and maximal intensity projection images of the z-stack were obtained using ZEN Blue (version 3.1).

### Viral vectors and oligonucleotides

LV sgRNA expression vectors were generated using pLentiCRISPRv1 (Addgene plasmid no. 49535) or pLentiCRISPRv2 (Addgene plasmid no. 52961). All LV vectors, sgRNAs, and oligonucleotide sequences used in this study are listed in Fig. S2 E; and Tables S1 and S2, respectively.

### LV production and B cell infection

Lenti-X 293T LV packaging cells (632180; Takara) were cultured in Dulbecco’s modified Eagle’s medium (Sigma-Aldrich) supplemented with 10% FBS, L-glutamine (4 mM, Gibco), sodium pyruvate (1 mM, Sigma-Aldrich), and penicillin/streptomycin (Gibco). The cells were maintained at 37°C with 5% CO_2_ and routinely tested for *Mycoplasma* contamination. Semi-confluent Lenti-X cells were cotransfected with LV plasmids, pCMVR8.74 helper (Addgene plasmid no. 22036), and pCMV-Eco (Cell Biolabs) envelope plasmids using polyethyleneimine (PEI) transfection (MW 25,000, Polysciences), as previously described (Michlits et al., 2020). B cell medium was used for LV collection. The virus-containing supernatant was cleared of cellular debris by centrifugation or filtration through a 0.45-μm PES filter before use for B cell transduction.

B cells were transduced at a density of 2 × 10^6^ cells per well in a 6-well plate with the ecotropic LV by spinfection at 800×*g* for 1 h at 32°C or 2 h at room temperature, in the presence of 4 μg/ml polybrene (Sigma-Aldrich). Immediately after spinfection, sBAFF and OP9 cells were added to the wells, and 16 h later, ∼80% of the medium in the well was carefully removed and fresh B cell medium, supplemented with sBAFF, was added. B cells were cultured for 3 days, and the percentage of transduction was analyzed. On average, 8% of the naïve B cells were transduced upon LV infection (Fig. 2 B), which is below the threshold recommended for achieving predominantly single-copy transduction (Doench, 2018; Michlits et al., 2020).

### Transfer of the transduced B cells

CD43^–^ B cells from the spleen and lymph nodes of donor mice were transduced with a mCherry-LV expressing a control sgRNA (sg.*Chr1*) by spinfection followed by subsequent culture for 3 days on OP9 cells with sBAFF, as described above. On the third day of culture, the transduced mCherry^+^ donor cells were sorted, and 200,000 sorted cells were transferred intravenously into recipient mice. Recipient mice were immunized with NP-KLH (in alum) or NP-Ficoll (in PBS) after 16 h, and the donor-derived mCherry^+^ cells from the spleen were analyzed by flow cytometry on different days after immunization (day 3, 5, 7, and 10; Fig. 3 B and Fig. S1 D). The percentages and numbers of total mCherry^+^ cells, mCherry^+^ plasmablasts, and mCherry^+^ GC B cells were analyzed. For proof-of-principle experiments, donor B cells were transduced with *Prdm1*- or *Irf4*-targeting sgRNAs, sorted, and transferred to recipient mice. Donor-derived mCherry^+^ cells were analyzed 6 days after immunization in recipient mice (Fig. 3 C).

### LV sgRNA library construction

The selection of the 379 genes for this study, which are highly expressed in plasmablasts compared with naïve B cells, is described in Fig. S1 E. The mouse sgRNA library was designed to target these 379 genes, with two sgRNAs per gene. Additionally, 112 sgRNAs were included to target 56 olfactory receptor genes, which were not expressed in the B cell lineage, along with 12 sgRNAs targeting essential genes (*Prc1*, *Cdk1, Top2a, Dtl, Chek1*, *and Espl1*). sgRNAs that induced full protein loss due to frameshift mutations were selected, as previously described (Michlits et al., 2020). In total, the library contained 882 sgRNAs (Table S2). The sgRNA library was constructed by using a pool of synthesized oligonucleotides (Twist Bioscience). Library amplification and cloning into the library vector (Fig. S2 E) were performed as described previously (Michlits et al., 2020).

The low-complexity CRISPR library, consisting of two sgRNAs per gene, is a key feature that enables *in vivo* CRISPR screens. To ensure efficient CRISPR-based knockout perturbations, we designed our sgRNA library based on the VBC score, an advanced sgRNA prediction algorithm, which reliably identifies sgRNAs that efficiently generate loss-of-function alleles (Michlits et al., 2020). The VBC score algorithm takes into account all the different steps in CRISPR/Cas9-based mutagenesis, i.e., DNA cleavage, repair outcome, impact on target proteins, and stringent selection against off-target effects. Notably, beyond the validations described in the original study, the VBC score has recently been independently validated as a superior sgRNA prediction algorithm by the Functional Genomics Consortium in a comprehensive analysis of different CRISPR libraries and sgRNA design strategies (Lukasiak et al., 2025).

### Analysis of editing efficiency of sgRNAs

Mature CD43^–^ B cells were infected with a single sgRNA-expressing mCherry-LV, cultured for 3 days on OP9 cells with sBAFF, and then stimulated with CpG, IL-4, and IL-5 for another 3 days, as described above (*in vitro* B cell cultures). The infected mCherry^+^ B cells were isolated by flow cytometric sorting, followed by DNA preparation. Indel sequencing was performed by PCR amplification (Table S1) and sequencing of a DNA fragment spanning the sgRNA break site, and the editing efficiency of the sgRNA was determined by the TIDE analysis (Brinkman et al., 2014).

### *In vivo* CRISPR/Cas9 screening system

For screening, the sgRNA library was packaged into LV particles by PEI transfection in Lenti-X 293T cells (632180; Takara). The virus-containing supernatant was cleared of cellular debris by filtration through a 0.45-μm PES filter and used to transduce B cells. Donor B cells were enriched by immunomagnetic depletion of non-B cells using CD43 MicroBeads from the spleens and lymph nodes of CD45.2 *Rosa26*^LSL-Cas9/LSL-EcoR^*Igk*^–/–^*Igh*^B1-8hi/+^*Cd23*-Cre mice. Infected mCherry^+^ B cells were sorted 3 days after transduction, and two aliquots of 240,000 cells (∼300-fold cell coverage per sgRNA) were pelleted, stored at −80°C, and saved as donor B cell input controls. Transduced mCherry^+^ donor B cells, 200,000 cells per recipient, were intravenously transferred to CD45.2/CD45.1 *Cd23*-Cre *Rosa26*^Cas9/+^ recipient mice.

In the morning after the transfer of donor B cells, recipient mice were immunized with NP-KLH in alum (TD screen). 7 days after immunization, the spleens were dissected, and splenic single-cell suspensions were prepared. Red blood cells were lysed with ACK buffer (Gibco), and the cell suspension was incubated with anti-CD16/32 antibody at 1 μg/ml in FACS buffer for 15 min. Donor-derived cells were then enriched by further incubation of the cell suspension with biotinylated anti-CD45.1 antibody at a final concentration of 1 μg/ml and subsequent immunomagnetic depletion of CD45.1^+^ cells with Anti-Biotin MicroBeads (Miltenyi Biotec). CD45.2^+^-enriched donor-derived B cells were stained with antibodies, and CD138^+^TACI^+^ plasmablasts and CD19^+^GL7^+^CD95^+^ GC B cells were sorted. The gating strategy used for flow cytometric cell sorting is shown in Fig. S3 B. Sorted plasmablasts and GC B cells from each recipient mouse were pelleted and frozen at −80°C until further processing. The screening experiments were performed twice. A total of 21 recipients were divided into four groups analyzed as biological replicate samples (Fig. S3 D).

For the screening performed upon induction of a TI B cell response, donor B cells were isolated and transferred to recipient mice, as described above. Fractions of 200,000 mCherry^+^-transduced donor B cells were used as the donor B cell input controls. Recipient mice were immunized the next morning with NP-Ficoll (in PBS), and their spleens were dissected 6 or 7 days after immunization. CD45.2^+^-enriched donor-derived B cells were stained with antibodies, and CD138^+^TACI^+^ plasmablasts and CD138^–^TACI^+^ memory B cells were sorted. The gating strategy used for flow cytometric cell sorting is shown in Fig. S4 B. Sorted plasmablasts and CD138^–^TACI^+^ cells from each recipient mouse were pelleted and frozen at −80°C until further processing. The screening experiment was performed twice, as described above. 25 recipients were divided into three groups, which were analyzed as biological replicate samples (Fig. S4 D).

### Generation of next-generation sequencing libraries

Next-generation sequencing (NGS) libraries of sorted cells were prepared as previously described (Michlits et al., 2020). Briefly, genomic DNA was isolated by cell lysis (10 mM Tris-HCl, 150 mM NaCl, 10 mM EDTA, 0.1% SDS), proteinase K treatment, and digestion with DNase-free RNase (10977035; Thermo Fisher Scientific). Lysates from sorted mouse cells in the same experiment were pooled to achieve a representation of 500–900 cells per sgRNA for TD B cell responses (Fig. S3 D) or ∼100 cells per sgRNA for TI B cell responses (Fig. S4 D). Four or three replicate samples were prepared for each cell type.

Genomic DNA was isolated using two rounds of phenol extraction and isopropanol precipitation. Genomic DNA was subjected to several freeze–thaw cycles before nested PCR amplification of the sgRNA cassette. Barcoded NGS libraries were generated for each pooled sample by using a two-step PCR protocol. The first PCR used 0.5 μl of Q5 Hot Start High-Fidelity DNA Polymerase (M0493; New England Biolabs) in 50-μl reactions containing 100 ng of genomic DNA. For each sample, the resulting PCR products were pooled and purified using MBSpure magnetic PCR purification beads (in-house) and used as input for a second PCR, introducing standard Illumina adapters using 10 ng of the DNA template. The final Illumina libraries were pooled and sequenced using a HiSeq 2500 platform (Illumina). Primers used for library amplification are listed in Table S1.

### Bioinformatics analysis of the pooled sgRNA-sequencing data

To quantify raw sequencing reads, we used the crispr-process-nf Nextflow pipeline, available at https://github.com/ZuberLab/crispr-process-nf as described previously (de Almeida et al., 2021). In brief, all guides in the sgRNA library were padded with Cs to equal length before creating an index for Bowtie 2 (version 2.3.0). Random 6mer nucleotides were trimmed using the fastx_trimmer from the fastx-toolkit (version 0.0.14) (http://hannonlab.cshl.edu/fastx_toolkit/) before demultiplexing using 4mer sample barcodes with a fastx_barcode splitter (--mismatches 1 --bol). Next, barcodes and 20mer spacers were trimmed, and reads were aligned with Bowtie 2 and quantified with featureCounts (version 1.6.1). To calculate the enrichment or depletion of sgRNAs, we pooled the data of the two experiments, containing both replicate samples generated for each cell type (Fig. S3 D and Fig. S4 D). To this end, we used the crispr-mageck-nf Nextflow workflow, available at https://github.com/ZuberLab/crispr-mageck-nf. First, count tables were filtered to exclude sgRNAs with fewer than 50 counts in the control and sorted samples before further downstream analyses. Read counts were median-normalized, and average log_2_ fold changes, P values, and false discovery rates were calculated using MAGeCK (0.5.9) (Li et al., 2014). If the sorted samples had a median of 0, a +1 pseudocount was added to each sgRNA for each sample included in the analysis. To calculate the enrichment of sgRNAs in sorted plasmablasts, GC B cells, and memory B cells, sgRNA counts within the sorted populations were compared with sorted donor B cell populations. For the analysis of gene hits, sgRNAs were ranked according to their abundance in the donor B cell control samples (Table S3). For TD responses, lowly abundant sgRNAs with a rank value below 70 and, for TI responses, sgRNAs with a rank value below 106 were excluded. A group of genes with an essentiality score lower than −0.2 (*Cenpi, Crls1, Ddost, Hsp90b1, Hspa5, Odc1, Ppa1, Rpn1, Sec61g, Sec63, Sel1l, Slc33a1, Slc35b1, Spcs2, Spcs3, Uba5*) were also excluded from the list of gene hits due to their essential functions in different cell types (Table S3).

### Validation of potential positive and negative regulators

To validate the gene hits of the screens, naïve B cells were isolated from the spleen of *Cd23*-Cre *Rosa26*^LSL-Cas9/LSL-EcoR^*Igk*^–/–^*Igh*^B1-8hi/+^ donor mice and transduced with ecotropic LV particles expressing a sgRNA targeting the gene to be validated or a neutral control sgRNA (sg.*Chr1*), targeting a sequence in a gene desert region of chromosome 1 (Table S1), and expressing the mCherry fluorescent reporter protein. In parallel, a fraction of naïve B cells was transduced with LV particles expressing the control sg.*Chr1* and mAmetrine fluorescent protein. After spinfection and 3 days of *in vitro* culture, the transduced mCherry^+^ and mAmetrine^+^ donor B cells were sorted by flow cytometry, mixed at a 2.5:1 ratio, and transferred to recipient mice. Control mice received a mixture of mCherry^+^ control sg.*Chr1* B cells and mAmetrine^+^ sg.*Chr1* B cells. The experimental recipient mice received a mixture of mCherry^+^ B cells expressing the sgRNA targeting the test gene, and mAmetrine^+^ sg.*Chr1* B cells. Control and experimental recipient mice received ∼200,000 total donor B cells by intravenous injection. Recipient mice were immunized, and 7 days after immunization, mCherry^+^ and mAmetrine^+^ donor-derived B cells in the spleen were analyzed by flow cytometry. The percentages of mCherry^+^ plasmablasts or mCherry^+^ GC B cells were determined relative to the percentages of mAmetrine^+^ plasmablasts or mAmetrine^+^ GC B cells, respectively, as shown in Fig. 6, B and C, and described in Fig. S5 C.

We mixed the experimental mCherry^+^ and control mAmetrine^+^ sgRNA-transduced B cells at a ratio of 2.5:1 prior to their transfer into recipient mice based on the following two reasons. First, the infection efficiency of the mCherry-LV was higher than that of the mAmetrine-LV, which thus resulted in higher numbers of infected mCherry^+^ B cells compared with the infected mAmetrine^+^ B cells. Consequently, a higher number of the important experimental mCherry^+^ B cells could be analyzed in the validation experiments. Second, we used the mAmetrine^+^ B cells only as control cells to calculate and normalize the effect of the sgRNAs of interest in the mCherry^+^ B cells among the different transplanted mice as described in Fig. S5 C.

### Statistical analysis

Statistical analyses were performed using the GraphPad Prism 10 software. Two-tailed unpaired Student’s *t* test was used to assess the statistical significance of one observed parameter between two experimental groups. Holm–Šídák’s correction test was employed, when multiple unpaired Student’s *t* tests were applied. When more than two experimental groups were compared, one-way analysis of variance was used combined with Tukey’s multiple comparisons tests to determine the statistical significance.

### Online supplemental material

Fig. S1 describes the analysis of different aspects of the *in vivo* sgRNA screening system. Fig. S2 contains the experimental data that determined the editing efficiency of selected sgRNAs, identified GC B cell– and plasmablast-specific sgRNA hits, and identified the sgRNA representations at different stage of the TD and TI screening experiments. Moreover, schematic diagrams of the different LV vectors used are shown. Fig. S3 contains a schematic diagram, the flow cytometric sorting data, and the sgRNA representation results of the *in vivo* screening experiments that identified novel regulators of the TD B cell responses. Fig. S4 contains a schematic diagram, the flow cytometric sorting data, and sgRNA representation results of the *in vivo* screening experiments that identified novel regulators of the TI B cell responses. Fig. S5 displays the validation data of gene hits of the TD B cell responses and contains an explanation how the validation data were normalized for their presentation in Fig. 6, B and C. Table S1 contains the sequences of the oligonucleotides used for gene cloning, library preparation, and PCR amplification. Table S2 contains the information and sequences of all sgRNAs used for the screening experiments. Table S3 contains the entire dataset of all sgRNA screening experiments in response to TD and TI immunization, including the normalized read counts, fold changes, and P values of all sgRNAs.

## Supplementary Material

Table S1 contains the sequences of the oligonucleotides used for gene cloning, library preparation, and PCR amplification.

Table S2 contains the information and sequences of all sgRNAs used for the screening experiments.

Table S3 contains the entire dataset of all sgRNA screening experiments in response to TD and TI immunization, including the normalized read counts, fold changes, and P values of all sgRNAs.

## Data Availability

The data of the sgRNA CRISPR/Cas9 screening experiments, underlying Fig. 4, are available in Table S3 in the online supplemental material of the published article.

## References

[bib1] Abdollahpour, H., G. Appaswamy, D. Kotlarz, J. Diestelhorst, R. Beier, A.A. Schäffer, E.M. Gertz, A. Schambach, H.H. Kreipe, D. Pfeifer, . 2012. The phenotype of human STK4 deficiency. Blood. 119:3450–3457. 10.1182/blood-2011-09-378158 22294732 PMC3325036

[bib2] de Almeida, M., M. Hinterndorfer, H. Brunner, I. Grishkovskaya, K. Singh, A. Schleiffer, J. Jude, S. Deswal, R. Kalis, M. Vunjak, . 2021. AKIRIN2 controls the nuclear import of proteasomes in vertebrates. Nature. 599:491–496. 10.1038/s41586-021-04035-8 34711951

[bib3] Ardestani, A., B. Lupse, and K. Maedler. 2018. Hippo signaling: Key emerging pathway in cellular and whole-body metabolism. Trends Endocrinol. Metab. 29:492–509. 10.1016/j.tem.2018.04.006 29739703

[bib4] Bai, X., L. Huang, L. Niu, Y. Zhang, J. Wang, X. Sun, H. Jiang, Z. Zhang, H. Miller, W. Tao, . 2016. Mst1 positively regulates B-cell receptor signaling via CD19 transcriptional levels. Blood Adv. 1:219–230. 10.1182/bloodadvances.2016000588 29296937 PMC5737167

[bib5] Barreira da Silva, R., R.M. Leitao, X. Pechuan-Jorge, S. Werneke, J. Oeh, V. Javinal, Y. Wang, W. Phung, C. Everett, J. Nonomiya, . 2022. Loss of the intracellular enzyme QPCTL limits chemokine function and reshapes myeloid infiltration to augment tumor immunity. Nat. Immunol. 23:568–580. 10.1038/s41590-022-01153-x 35314846

[bib6] Bettigole, S.E., and L.H. Glimcher. 2015. Endoplasmic reticulum stress in immunity. Annu. Rev. Immunol. 33:107–138. 10.1146/annurev-immunol-032414-112116 25493331

[bib7] Bock, C., P. Datlinger, F. Chardon, M.A. Coelho, M.B. Dong, K.A. Lawson, T. Lu, L. Maroc, T.M. Norman, B. Song, . 2022. High-content CRISPR screening. Nat. Rev. Methods Primers. 2:1–23. 10.1038/s43586-021-00093-4 PMC10200264 37214176

[bib8] Bradl, H., W. Schuh, and H.-M. Jäck. 2004. CD44 is dispensable for B lymphopoiesis. Immunol. Lett. 95:71–75. 10.1016/j.imlet.2004.06.004 15325800

[bib9] Brinkman, E.K., T. Chen, M. Amendola, and B. van Steensel. 2014. Easy quantitative assessment of genome editing by sequence trace decomposition. Nucleic Acids Res. 42:e168. 10.1093/nar/gku936 25300484 PMC4267669

[bib10] Cassese, G., S. Arce, A.E. Hauser, K. Lehnert, B. Moewes, M. Mostarac, G. Muehlinghaus, M. Szyska, A. Radbruch, and R.A. Manz. 2003. Plasma cell survival is mediated by synergistic effects of cytokines and adhesion-dependent signals1. J. Immunol. 171:1684–1690. 10.4049/jimmunol.171.4.1684 12902466

[bib11] Choi, J., T.M. Baldwin, M. Wong, J.E. Bolden, K.A. Fairfax, E.C. Lucas, R. Cole, C. Biben, C. Morgan, K.A. Ramsay, . 2019. Haemopedia RNA-seq: A database of gene expression during haematopoiesis in mice and humans. Nucleic Acids Res. 47:D780–D785. 10.1093/nar/gky1020 30395284 PMC6324085

[bib12] Chu, V.T., R. Graf, T. Wirtz, T. Weber, J. Favret, X. Li, K. Petsch, N.T. Tran, M.H. Sieweke, C. Berek, . 2016. Efficient CRISPR-mediated mutagenesis in primary immune cells using CrispRGold and a C57BL/6 Cas9 transgenic mouse line. Proc. Natl. Acad. Sci. USA. 113:12514–12519. 10.1073/pnas.1613884113 27729526 PMC5098665

[bib13] Closs, E.I., J.-P. Boissel, A. Habermeier, and A. Rotmann. 2006. Structure and function of cationic Amino Acid Transporters (CATs). J. Membr. Biol. 213:67–77. 10.1007/s00232-006-0875-7 17417706

[bib14] Cyster, J.G., and C.D.C. Allen. 2019. B cell responses: Cell interaction dynamics and decisions. Cell. 177:524–540. 10.1016/j.cell.2019.03.016 31002794 PMC6538279

[bib15] Devireddy, L.R., C. Gazin, X. Zhu, and M.R. Green. 2005. A cell-surface receptor for lipocalin 24p3 selectively mediates apoptosis and iron uptake. Cell. 123:1293–1305. 10.1016/j.cell.2005.10.027 16377569

[bib16] Didonna, A., E. Canto Puig, Q. Ma, A. Matsunaga, B. Ho, S.J. Caillier, H. Shams, N. Lee, S.L. Hauser, Q. Tan, . 2020. Ataxin-1 regulates B cell function and the severity of autoimmune experimental encephalomyelitis. Proc. Natl. Acad. Sci. 117:23742–23750. 10.1073/pnas.2003798117 32878998 PMC7519225

[bib17] Doench, J.G. 2018. Am I ready for CRISPR? A user’s guide to genetic screens. Nat. Rev. Genet. 19:67–80. 10.1038/nrg.2017.97 29199283

[bib18] Dubrot, J., P.P. Du, S.K. Lane-Reticker, E.A. Kessler, A.J. Muscato, A. Mehta, S.S. Freeman, P.M. Allen, K.E. Olander, K.M. Ockerman, . 2022. In vivo CRISPR screens reveal the landscape of immune evasion pathways across cancer. Nat. Immunol. 23:1495–1506. 10.1038/s41590-022-01315-x 36151395

[bib19] Elsner, R.A., and M.J. Shlomchik. 2020. Germinal center and extrafollicular B cell responses in vaccination, immunity, and autoimmunity. Immunity. 53:1136–1150. 10.1016/j.immuni.2020.11.006 33326765 PMC7748291

[bib20] Fang, C., T.D. Manes, L. Liu, K. Liu, L. Qin, G. Li, Z. Tobiasova, N.C. Kirkiles-Smith, M. Patel, J. Merola, . 2019. ZFYVE21 is a complement-induced Rab5 effector that activates non-canonical NF-κB via phosphoinosotide remodeling of endosomes. Nat. Commun. 10:2247. 10.1038/s41467-019-10041-2 31113953 PMC6529429

[bib21] Fooksman, D.R., Z. Jing, and R. Park. 2024. New insights into the ontogeny, diversity, maturation and survival of long-lived plasma cells. Nat. Rev. Immunol. 24:461–470. 10.1038/s41577-024-00991-0 38332373

[bib22] Fu, G., C.S. Guy, N.M. Chapman, G. Palacios, J. Wei, P. Zhou, L. Long, Y.-D. Wang, C. Qian, Y. Dhungana, . 2021. Metabolic control of TFH cells and humoral immunity by phosphatidylethanolamine. Nature. 595:724–729. 10.1038/s41586-021-03692-z 34234346 PMC8448202

[bib23] Fukuda, S., M. Sumii, Y. Masuda, M. Takahashi, N. Koike, J. Teishima, H. Yasumoto, T. Itamoto, T. Asahara, K. Dohi, and K. Kamiya. 2001. Murine and human *SDF2L1* is an endoplasmic reticulum stress-inducible gene and encodes a new member of the Pmt/rt protein family. Biochem. Biophysical Res. Commun. 280:407–414. 10.1006/bbrc.2000.4111 11162531

[bib24] García de Vinuesa, C., A. Gulbranson-Judge, M. Khan, P. O’Leary, M. Cascalho, M. Wabl, G.G.B. Klaus, M.J. Owen, and I.C.M. MacLennan. 1999a. Dendritic cells associated with plasmablast survival. Eur. J. Immunol. 29:3712–3721. 10.1002/(SICI)1521-4141(199911)29:11<3712::AID-IMMU3712>3.0.CO;2-P 10556827

[bib25] García de Vinuesa, C., P. O’Leary, D.M.-Y. Sze, K.-M. Toellner, and I.C.M. MacLennan. 1999b. T-independent type 2 antigens induce B cell proliferation in multiple splenic sites, but exponential growth is confined to extrafollicular foci. Eur. J. Immunol. 29:1314–1323. 10.1002/(SICI)1521-4141(199904)29:04<1314::AID-IMMU1314>3.0.CO;2-4 10229099

[bib26] Glaros, V., R. Rauschmeier, A.V. Artemov, A. Reinhardt, S. Ols, A. Emmanouilidi, C. Gustafsson, Y. You, C. Mirabello, Å.K. Björklund, . 2021. Limited access to antigen drives generation of early B cell memory while restraining the plasmablast response. Immunity. 54:2005–2023.e10. 10.1016/j.immuni.2021.08.017 34525339 PMC7612941

[bib27] Gloury, R., D. Zotos, M. Zuidscherwoude, F. Masson, Y. Liao, J. Hasbold, L.M. Corcoran, P.D. Hodgkin, G.T. Belz, W. Shi, . 2016. Dynamic changes in Id3 and E-protein activity orchestrate germinal center and plasma cell development. J. Exp. Med. 213:1095–1111. 10.1084/jem.20152003 27217539 PMC4886367

[bib28] Gu, B., E. Posfai, and J. Rossant. 2018. Efficient generation of targeted large insertions by microinjection into two-cell-stage mouse embryos. Nat. Biotechnol. 36:632–637. 10.1038/nbt.4166 29889212

[bib29] Hargreaves, D.C., P.L. Hyman, T.T. Lu, V.N. Ngo, A. Bidgol, G. Suzuki, Y.-R. Zou, D.R. Littman, and J.G. Cyster. 2001. A coordinated change in chemokine responsiveness guides plasma cell movements. J. Exp. Med. 194:45–56. 10.1084/jem.194.1.45 11435471 PMC2193440

[bib30] Hong, L., X. Li, D. Zhou, J. Geng, and L. Chen. 2018. Role of Hippo signaling in regulating immunity. Cell Mol Immunol. 15:1003–1009. 10.1038/s41423-018-0007-1 29568120 PMC6269503

[bib31] Huang, B., J.D. Phelan, S. Preite, J. Gomez-Rodriguez, K.H. Johansen, H. Shibata, A.L. Shaffer, Q. Xu, B. Jeffrey, M. Kirby, . 2022. In vivo CRISPR screens reveal a HIF-1α-mTOR-network regulates T follicular helper versus Th1 cells. Nat. Commun. 13:805. 10.1038/s41467-022-28378-6 35145086 PMC8831505

[bib32] Huang, H., P. Zhou, J. Wei, L. Long, H. Shi, Y. Dhungana, N.M. Chapman, G. Fu, J. Saravia, J.L. Raynor, . 2021. In vivo CRISPR screening reveals nutrient signaling processes underpinning CD8+ T cell fate decisions. Cell. 184:1245–1261.e21. 10.1016/j.cell.2021.02.021 33636132 PMC8101447

[bib33] Janssens, W., M.K.L. Chuah, L. Naldini, A. Follenzi, D. Collen, J.-M. Saint-Remy, and T. VandenDriessche. 2003. Efficiency of Onco-retroviral and lentiviral gene transfer into primary mouse and human B-Lymphocytes is pseudotype dependent. Hum. Gene Ther. 14:263–276. 10.1089/10430340360535814 12639306

[bib34] Klein, U., S. Casola, G. Cattoretti, Q. Shen, M. Lia, T. Mo, T. Ludwig, K. Rajewsky, and R. Dalla-Favera. 2006. Transcription factor IRF4 controls plasma cell differentiation and class-switch recombination. Nat. Immunol. 7:773–782. 10.1038/ni1357 16767092

[bib35] Kuja-Panula, J., M. Kiiltomäki, T. Yamashiro, A. Rouhiainen, and H. Rauvala. 2003. AMIGO, a transmembrane protein implicated in axon tract development, defines a novel protein family with leucine-rich repeats. J. Cell Biol. 160:963–973. 10.1083/jcb.200209074 12629050 PMC2173769

[bib36] Kwon, K., C. Hutter, Q. Sun, I. Bilic, C. Cobaleda, S. Malin, and M. Busslinger. 2008. Instructive role of the transcription factor E2A in Early B lymphopoiesis and germinal center B cell development. Immunity. 28:751–762. 10.1016/j.immuni.2008.04.014 18538592

[bib37] Lam, Y.C., A.B. Bowman, P. Jafar-Nejad, J. Lim, R. Richman, J.D. Fryer, E.D. Hyun, L.A. Duvick, H.T. Orr, J. Botas, and H.Y. Zoghbi. 2006. ATAXIN-1 interacts with the repressor Capicua in its native complex to cause SCA1 neuropathology. Cell. 127:1335–1347. 10.1016/j.cell.2006.11.038 17190598

[bib38] Li, W., H. Xu, T. Xiao, L. Cong, M.I. Love, F. Zhang, R.A. Irizarry, J.S. Liu, M. Brown, and X.S. Liu. 2014. MAGeCK enables robust identification of essential genes from genome-scale CRISPR/Cas9 knockout screens. Genome Biol. 15:554. 10.1186/s13059-014-0554-4 25476604 PMC4290824

[bib39] Long, L., J. Wei, S.A. Lim, J.L. Raynor, H. Shi, J.P. Connelly, H. Wang, C. Guy, B. Xie, N.M. Chapman, . 2021. CRISPR screens unveil signal hubs for nutrient licensing of T cell immunity. Nature. 600:308–313. 10.1038/s41586-021-04109-7 34795452 PMC8887674

[bib40] Lukasiak, S., A. Kalinka, N. Gupta, A. Papadopoulos, K. Saeed, U. McDermott, G.J. Hannon, D. Ross-Thriepland, and D. Walter. 2025. A benchmark comparison of CRISPRn guide-RNA design algorithms and generation of small single and dual-targeting libraries to boost screening efficiency. BMC Genomics. 26:198. 10.1186/s12864-025-11386-3 40011813 PMC11863645

[bib41] Michlits, G., J. Jude, M. Hinterndorfer, M. de Almeida, G. Vainorius, M. Hubmann, T. Neumann, A. Schleiffer, T.R. Burkard, M. Fellner, . 2020. Multilayered VBC score predicts sgRNAs that efficiently generate loss-of-function alleles. Nat. Methods. 17:708–716. 10.1038/s41592-020-0850-8 32514112

[bib42] Minnich, M., H. Tagoh, P. Bönelt, E. Axelsson, M. Fischer, B. Cebolla, A. Tarakhovsky, S.L. Nutt, M. Jaritz, and M. Busslinger. 2016. Multifunctional role of the transcription factor Blimp-1 in coordinating plasma cell differentiation. Nat. Immunol. 17:331–343. 10.1038/ni.3349 26779602 PMC5790184

[bib43] Mittrücker, H.-W., T. Matsuyama, A. Grossman, T.M. Kündig, J. Potter, A. Shahinian, A. Wakeham, B. Patterson, P.S. Ohashi, and T.W. Mak. 1997. Requirement for the transcription factor LSIRF/IRF4 for mature B and T lymphocyte function. Science. 275:540–543. 10.1126/science.275.5299.540 8999800

[bib44] Müller-Winkler, J., R. Mitter, J.C.F. Rappe, L. Vanes, E. Schweighoffer, H. Mohammadi, A. Wack, and V.L.J. Tybulewicz. 2020. Critical requirement for BCR, BAFF, and BAFFR in memory B cell survival. J. Exp. Med. 218:e20202057. 10.1084/jem.20191393 PMC7604764 33119032

[bib45] Nagano, M., D. Hoshino, T. Sakamoto, N. Kawasaki, N. Koshikawa, and M. Seiki. 2010. ZF21 protein regulates cell adhesion and motility*. J. Biol. Chem. 285:21013–21022. 10.1074/jbc.M110.106443 20439989 PMC2898296

[bib46] Newman, R., and P. Tolar. 2021. Chronic calcium signaling in IgE+ B cells limits plasma cell differentiation and survival. Immunity. 54:2756–2771.e10. 10.1016/j.immuni.2021.11.006 34879220

[bib47] Nutt, S.L., P.D. Hodgkin, D.M. Tarlinton, and L.M. Corcoran. 2015. The generation of antibody-secreting plasma cells. Nat. Rev. Immunol. 15:160–171. 10.1038/nri3795 25698678

[bib48] Obukhanych, T.V., and M.C. Nussenzweig. 2006. T-independent type II immune responses generate memory B cells. J. Exp. Med. 203:305–310. 10.1084/jem.20052036 16476769 PMC2118207

[bib49] Ochiai, K., M. Maienschein-Cline, G. Simonetti, J. Chen, R. Rosenthal, R. Brink, A.S. Chong, U. Klein, A.R. Dinner, H. Singh, and R. Sciammas. 2013. Transcriptional regulation of germinal center B and plasma cell fates by dynamical control of IRF4. Immunity. 38:918–929. 10.1016/j.immuni.2013.04.009 23684984 PMC3690549

[bib50] Ono, T., N. Sekino-Suzuki, Y. Kikkawa, H. Yonekawa, and S. Kawashima. 2003. Alivin 1, a novel neuronal activity-dependent gene, inhibits apoptosis and promotes survival of cerebellar granule neurons. J. Neurosci. 23:5887–5896. 10.1523/JNEUROSCI.23-13-05887.2003 12843293 PMC6741272

[bib51] Pinter, T., M. Fischer, M. Schäfer, M. Fellner, J. Jude, J. Zuber, M. Busslinger, and M. Wöhner. 2022. Comprehensive CRISPR-Cas9 screen identifies factors which are important for plasmablast development. Front. Immunol. 13:979606. 10.3389/fimmu.2022.979606 36189249 PMC9521597

[bib52] Platt, R.J., S. Chen, Y. Zhou, M.J. Yim, L. Swiech, H.R. Kempton, J.E. Dahlman, O. Parnas, T.M. Eisenhaure, M. Jovanovic, . 2014. CRISPR-Cas9 knockin mice for genome editing and cancer modeling. Cell. 159:440–455. 10.1016/j.cell.2014.09.014 25263330 PMC4265475

[bib53] Ponta, H., L. Sherman, and P.A. Herrlich. 2003. CD44: From adhesion molecules to signalling regulators. Nat. Rev. Mol. Cell Biol. 4:33–45. 10.1038/nrm1004 12511867

[bib54] Protin, U., T. Schweighoffer, W. Jochum, and F. Hilberg. 1999. CD44-Deficient mice develop normally with changes in subpopulations and recirculation of lymphocyte subsets1. J. Immunol. 163:4917–4923. 10.4049/jimmunol.163.9.4917 10528194

[bib55] Rafi, A., M. Nagarkatti, and P.S. Nagarkatti. 1997. Hyaluronate-CD44 interactions can induce murine B-Cell activation. Blood. 89:2901–2908. 10.1182/blood.V89.8.2901 9108410

[bib56] Rhee, S.G., H.A. Woo, I.S. Kil, and S.H. Bae. 2012. Peroxiredoxin functions as a peroxidase and a regulator and sensor of local peroxides*. J. Biol. Chem. 287:4403–4410. 10.1074/jbc.R111.283432 22147704 PMC3281607

[bib57] Robinson, M.J., R.H. Webster, and D.M. Tarlinton. 2020. How intrinsic and extrinsic regulators of plasma cell survival might intersect for durable humoral immunity. Immunological Rev. 296:87–103. 10.1111/imr.12895 32592168

[bib58] Rossi, G.R., M.R. Mautino, and R.A. Morgan. 2003. High-efficiency lentiviral vector-mediated gene transfer into murine macrophages and activated splenic B lymphocytes. Hum. Gene Ther. 14:385–391. 10.1089/104303403321208989 12659679

[bib59] Sasako, T., M. Ohsugi, N. Kubota, S. Itoh, Y. Okazaki, A. Terai, T. Kubota, S. Yamashita, K. Nakatsukasa, T. Kamura, . 2019. Hepatic Sdf2l1 controls feeding-induced ER stress and regulates metabolism. Nat. Commun. 10:947. 10.1038/s41467-019-08591-6 30814508 PMC6393527

[bib60] Sato, Y., R. Kojima, M. Okumura, M. Hagiwara, S. Masui, K. Maegawa, M. Saiki, T. Horibe, M. Suzuki, and K. Inaba. 2013. Synergistic cooperation of PDI family members in peroxiredoxin 4-driven oxidative protein folding. Sci. Rep. 3:2456. 10.1038/srep02456 23949117 PMC3744794

[bib61] Sciammas, R., A.L. Shaffer, J.H. Schatz, H. Zhao, L.M. Staudt, and H. Singh. 2006. Graded expression of interferon regulatory factor-4 coordinates isotype switching with plasma cell differentiation. Immunity. 25:225–236. 10.1016/j.immuni.2006.07.009 16919487

[bib62] Shapiro-Shelef, M., K.-I. Lin, L.J. McHeyzer-Williams, J. Liao, M.G. McHeyzer-Williams, and K. Calame. 2003. Blimp-1 is required for the formation of immunoglobulin secreting plasma cells and pre-plasma memory B cells. Immunity. 19:607–620. 10.1016/S1074-7613(03)00267-X 14563324

[bib63] Shi, W., Y. Liao, S.N. Willis, N. Taubenheim, M. Inouye, D.M. Tarlinton, G.K. Smyth, P.D. Hodgkin, S.L. Nutt, and L.M. Corcoran. 2015. Transcriptional profiling of mouse B cell terminal differentiation defines a signature for antibody-secreting plasma cells. Nat. Immunol. 16:663–673. 10.1038/ni.3154 25894659

[bib64] Shih, T.-A.Y., M. Roederer, and M.C. Nussenzweig. 2002. Role of antigen receptor affinity in T cell–independent antibody responses in vivo. Nat. Immunol. 3:399–406. 10.1038/ni776 11896394

[bib65] Stephan, A., M. Wermann, A. von Bohlen, B. Koch, H. Cynis, H.-U. Demuth, and S. Schilling. 2009. Mammalian glutaminyl cyclases and their isoenzymes have identical enzymatic characteristics. FEBS J. 276:6522–6536. 10.1111/j.1742-4658.2009.07337.x 19804409

[bib66] Tavender, T.J., A.M. Sheppard, and N.J. Bulleid. 2008. Peroxiredoxin IV is an endoplasmic reticulum-localized enzyme forming oligomeric complexes in human cells. Biochem. J. 411:191–199. 10.1042/BJ20071428 18052930 PMC4864507

[bib67] Taylor, J.J., K.A. Pape, H.R. Steach, and M.K. Jenkins. 2015. Apoptosis and antigen affinity limit effector cell differentiation of a single naïve B cell. Science. 347:784–787. 10.1126/science.aaa1342 25636798 PMC4412594

[bib68] Tellier, J., W. Shi, M. Minnich, Y. Liao, S. Crawford, G.K. Smyth, A. Kallies, M. Busslinger, and S.L. Nutt. 2016. Blimp-1 controls plasma cell function through the regulation of immunoglobulin secretion and the unfolded protein response. Nat. Immunol. 17:323–330. 10.1038/ni.3348 26779600 PMC4757736

[bib69] Tellier, J., I. Tarasova, J. Nie, C.S. Smillie, P.L. Fedele, W.H.J. Cao, J.R. Groom, G.T. Belz, D. Bhattacharya, G.K. Smyth, and S.L. Nutt. 2024. Unraveling the diversity and functions of tissue-resident plasma cells. Nat. Immunol. 25:330–342. 10.1038/s41590-023-01712-w 38172260

[bib70] Trezise, S., I.Y. Kong, E.D. Hawkins, M.J. Herold, S.N. Willis, and S.L. Nutt. 2023. An arrayed CRISPR screen of primary B cells reveals the essential elements of the antibody secretion pathway. Front. Immunol. 14:1089243. 10.3389/fimmu.2023.1089243 36860866 PMC9969136

[bib71] Turner, C.A., D.H. Mack, and M.M. Davis. 1994. Blimp-1, a novel zinc finger-containing protein that can drive the maturation of B lymphocytes into immunoglobulin-secreting cells. Cell. 77:297–306. 10.1016/0092-8674(94)90321-2 8168136

[bib72] Turner, D.J., A. Saveliev, F. Salerno, L.S. Matheson, M. Screen, H. Lawson, D. Wotherspoon, K.R. Kranc, and M. Turner. 2022. A functional screen of RNA binding proteins identifies genes that promote or limit the accumulation of CD138+ plasma cells. Elife. 11:e72313. 10.7554/eLife.72313 35451955 PMC9106329

[bib73] Victora, G.D., and M.C. Nussenzweig. 2022. Germinal centers. Annu. Rev. Immunol. 40:413–442. 10.1146/annurev-immunol-120419-022408 35113731

[bib74] Willis, S.N., K.L. Good-Jacobson, J. Curtis, A. Light, J. Tellier, W. Shi, G.K. Smyth, D.M. Tarlinton, G.T. Belz, L.M. Corcoran, . 2014. Transcription factor IRF4 regulates germinal center cell formation through a B cell–intrinsic mechanism. J. Immunol. 192:3200–3206. 10.4049/jimmunol.1303216 24591370

[bib75] Wöhner, M., T. Pinter, P. Bönelt, A. Hagelkruys, D. Kostanova-Poliakova, J. Stadlmann, S.F. Konieczny, M. Fischer, M. Jaritz, and M. Busslinger. 2022. The Xbp1-regulated transcription factor Mist1 restricts antibody secretion by restraining Blimp1 expression in plasma cells. Front. Immunol. 13:859598. 10.3389/fimmu.2022.859598 36618345 PMC9811352

[bib76] Wöhner, M., H. Tagoh, I. Bilic, M. Jaritz, D.K. Poliakova, M. Fischer, and M. Busslinger. 2016. Molecular functions of the transcription factors E2A and E2-2 in controlling germinal center B cell and plasma cell development. J. Exp. Med. 213:1201–1221. 10.1084/jem.20152002 27261530 PMC4925024

[bib77] Xiao, C., L. Srinivasan, D.P. Calado, H.C. Patterson, B. Zhang, J. Wang, J.M. Henderson, J.L. Kutok, and K. Rajewsky. 2008. Lymphoproliferative disease and autoimmunity in mice with increased miR-17-92 expression in lymphocytes. Nat. Immunol. 9:405–414. 10.1038/ni1575 18327259 PMC2533767

[bib78] Xiong, E., O. Popp, C. Salomon, P. Mertins, C. Kocks, K. Rajewsky, and V.T. Chu. 2023. A CRISPR/Cas9-mediated screen identifies determinants of early plasma cell differentiation. Front. Immunol. 13:1083119. 10.3389/fimmu.2022.1083119 36685499 PMC9849354

[bib79] Yan, Y., C. Wladyka, J. Fujii, and S. Sockanathan. 2015. Prdx4 is a compartment-specific H2O2 sensor that regulates neurogenesis by controlling surface expression of GDE2. Nat. Commun. 6:7006. 10.1038/ncomms8006 25943695 PMC4432624

[bib80] Yang, J., D. Goetz, J.-Y. Li, W. Wang, K. Mori, D. Setlik, T. Du, H. Erdjument-Bromage, P. Tempst, R. Strong, and J. Barasch. 2002. An iron delivery pathway mediated by a lipocalin. Mol. Cell. 10:1045–1056. 10.1016/S1097-2765(02)00710-4 12453413

[bib81] Yoshida, H., C.A. Lareau, R.N. Ramirez, S.A. Rose, B. Maier, A. Wroblewska, F. Desland, A. Chudnovskiy, A. Mortha, C. Dominguez, . 2019. The *cis*-regulatory atlas of the mouse immune system. Cell. 176:897–912.e20. 10.1016/j.cell.2018.12.036 30686579 PMC6785993

[bib82] Zou, Y.R., S. Takeda, and K. Rajewsky. 1993. Gene targeting in the Ig kappa locus: Efficient generation of lambda chain‐expressing B cells, independent of gene rearrangements in Ig kappa. EMBO J. 12:811–820. 10.1002/j.1460-2075.1993.tb05721.x 8458339 PMC413278

